# Synthesis, characterization, COX1/2 inhibition and molecular modeling studies on novel 2-thio-diarylimidazoles

**DOI:** 10.3906/kim-2104-54

**Published:** 2021-08-06

**Authors:** Zafer ŞAHİN, Melike KOÇOĞLU KALKAN, Barkın BERK, Leyla YURTTAŞ, Ceysu BENDER, Sevde Nur BİLTEKİN KALELİ, Şeref DEMİRAYAK

**Affiliations:** 1 Department of Pharmaceutical Chemistry, School of Pharmacy, İstanbul Medipol University, İstanbul, Turkey; 2 Department of Chemistry, Faculty of Science, Ankara University, Ankara, Turkey; 3 Department of Pharmaceutical Chemistry, Faculty of Pharmacy, Anadolu University, Eskişehir, Turkey; 4 Department of Pharmaceutical Microbiology, School of Pharmacy, İstanbul Medipol University, İstanbul, Turkey

**Keywords:** Imidazole, COX-1, COX-2, molecular modeling, inflammation

## Abstract

Heterocyclic compounds with diaryl substituents have been a milestone approach for selective cyclooxygenase 2 (COX 2) inhibition by bioisosteric replacements and modifications. It is also known that thiazole derivatives have different pharmacological activities. In this study, nine novel 2-[(1,5-diphenyl-1*H*-imidazole-2-yl)thio]-N-(thiazole-2-yl)acetamide derivatives (Compound **1–9**) were synthesized via the reaction of 1,5-disubstitued phenyl-imidazole-2-thiole and *N*-thiazole acetamide. The inhibitory effects of COX-1 and COX-2 enzymes were tested for the synthesized compounds. Enzyme-ligand interactions of the most active compound on COX subtypes were elucidated by molecular modeling studies. The percent inhibitory effect for compound **1**, which is the naked derivative among all the compounds, was found to be the most active on COX-2 at 10 μM concentration (**C1**_COX-2_: 88%, SC-560_COX-2_: 98.2%, **C1**_COX-1_: 60.9%); whereas compound **9** showed the highest inhibitory effect and was found to be the most selective derivative on COX-1 isoenzyme (**C9**_COX-1_: 85%, DuP-697_COX-1_: 97.2%, **C9**_COX-2_: 57.9%)

## 1. Introduction

Inflammation is known to be the response of live tissues to suffer and in the following process many changes occur within the cell. Some of these changes are; enzyme induction, inflammatory mediator release, fluid cumulation, cell migration, tissue deterioration and repair. Synthesis of prostaglandins is generally increased in the inflammation zone. Prostaglandins (PGE2, PGI2, PGF2a), prostacyclin (PGI2) and thromboxanes (TXA2), which are proinflammatory chemicals, are triggered by phospholipase A2, and it causes the release of arachidonic acid, which is an essential mediator and substrate for cyclooxygenases. An antiinflammatory effect is observed by the inhibition of cyclooxygenases (COX) that catalyze the conversion of arachidonic acid to prostaglandins. The pharmacotherapy with nonsteroidal antiinflammatory drugs (NSAIDs) for inflammation and pain, often leads to adverse side effects that effects gastrointestinal system and kidneys [1–3].

Currently, two isoforms of COX enzyme is known; COX-1 and COX-2 [1,2]. The constitutive COX-1 isoenzyme is excreted in a variety of tissues and is essential for maintaining homeostatic functions such as stomach mucosal protection and vascular systems. Alternatively, the COX-2 subtype is induced by immunologic stimulation that links its participation in inflammatory responses [4]. Therefore, more particular inhibition of COX-2 than COX-1 is necessary in the symptomatic therapy of inflammation and related diseases with altered gastrointestinal adverse effects compared to NSAIDs. In addition to rheumatoid arthritis and osteoarthritis, COX-2 plays a major role in colorectal carcinogenesis and angiogenesis [5,6]. Some studies, in recent years, have revealed that the progression of Alzheimer’s disease (AD) is decreasing among some NSAID users. Therefore, continuous therapy with COX-2 selective inhibitors slows down the progression of AD without causing gastrointestinal damage [7]. For this reason, potent and highly selective COX-2 inhibitors have been designed as novel trend NSAIDs with altered gastrointestinal adverse effects. Nevertheless, high COX-2 selective drugs valdecoxib and rofecoxib have been withdrawn from the market due to the increased cardiovascular adverse symptoms. Prostacyclin regulates the synthesis of a vasodilator and platelet aggregation inhibitors. Inhibition of prostacyclin production by selective COX-2 inhibitors explains its adverse cardiovascular effects [8,9]. Therefore, compounds having low selectivity for COX-2 are sought to reduce probable cardiovascular adverse effects.

**Figure 1:**
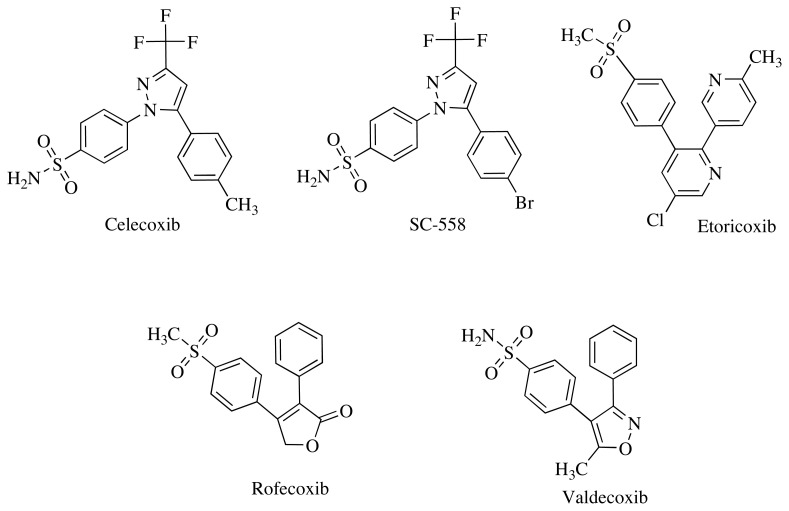
COX inhibitors with five-membered rings bearing diaryl substituents.

Diaryl substituted heterocyclic compounds which usually have five-membered pharmacophore parent structures have been largely developed as more selective COX-2 enzyme inhibitors. All the mentioned compounds have been formed by diaryl substitution in the central ring, which is usually a heterocycle (Figure 1) [10].

In this study, 9 novel 2-[(1,5-disubstituted phenyl-1*H*-imidazol-2-yl) thio]-*N*- (thiazol-2-yl) acetamide derivatives have been defined to develop new type of COX inhibitors with moderate inhibitory activity.

## 2. Materials and methods

### 2.1. Synthesis

Chemicals used in synthesis and activity were purchased from Aldrich Chemical Co. (Steinheim, Germany). Melting points were recorded with a MP90 digital melting point apparatus (Mettler Toledo, Columbus). IR spectra (KBr) were recorded on a Shimadzu, IR Affinity-1 S (Shimadzu, Japan) and ^1^H NMR spectra were obtained by Bruker 300 MHz FT-NMR Spectrometer (Bruker Bioscience, Billerica, MA) (Bruker Bioscience, Billerica, MA). ^13^C NMR spectra were measured using a Bruker 75 MHz FT-NMR Spectrometer. All the chemical shift values were recorded as δ (ppm). Mass spectra were obtained using an Shimadzu LCMS-IT-TOF system (Shimadzu, Tokyo, Japan). The completion of reactions and purity of the synthesized final compounds were controlled by thin-layer chromatography (TLC) on silica gel-coated aluminum sheets. Elemental analyses were performed with a LECO CHNS 932 analyzer (Leco Corp., MI, USA). Enzyme assay results were measured in Multimode Plate Reader (Molecular Devices, Spectramax i3x (US). Molecular modeling studies were performed in GLIDE Docking mode of Schrodinger Maestro program (Schrodinger Inc, ABD).

### 2.2. General synthesis methods

#### 2.2.1. Synthesis of 2-amino-4′-substituted acetophenone hydrochloride derivatives (Method A)

**Figure 2:**
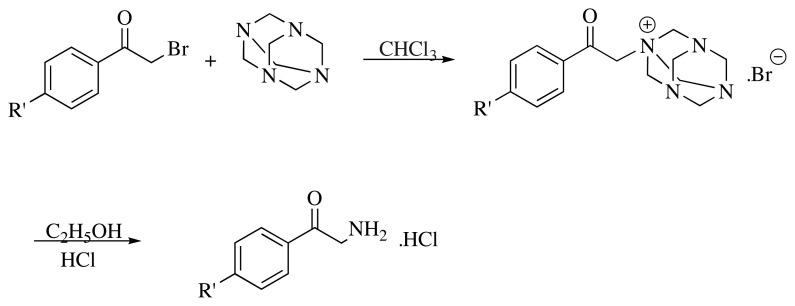
Synthesis of 2-amino-4′-substituted acetophenone hydrochloride derivatives.

Hexamethylenetetramine (urotropine) (0.67 mol) was stirred with chloroform for a few minutes at room temperature under continuous temperature control at 30 °C. Then, 2-bromo-4′-substituted acetophenone derivative was added in portions. The mixture was heated to 50–52 °C and stirred for 4 h and then solvent removed by filtration at 30 °C. The solid was washed with chloroform and dried in an oven. Afterwards, it was stirred in 95% ethanol (175 mL) and concentrated HCl (85 mL) mixture, solid was dissolved firstly, after stirring for 16 h, HCl salt of compound was precipitated, and solvent was removed by filtration. Solid was dried in a vacuum oven (Figure 2) [11].

#### 2.2.2. Synthesis of 1,5-Disubstituted phenyl-1*H*-imidazole-2-thiol derivatives (Method B)

**Figure 3:**
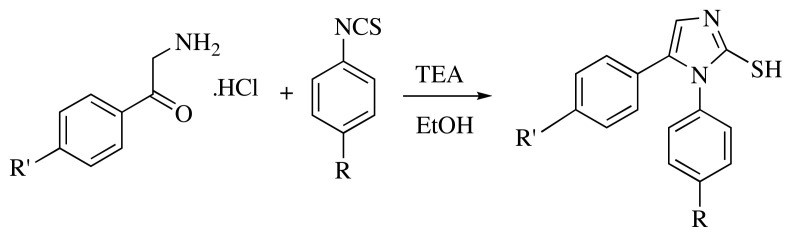
Synthesis of 1*H*-imidazole-2-thiol derivatives.

Method B was carried out by modifying the Markwald synthesis procedure. 2-Amino-1- (4′-substituted phenyl) ethan-1-one hydrochloride derivative (0.025 mol) and 4-substituted phenyl isothiocyanate derivative (0.025 mol) was stirred in ethanol (30–50 mL) while triethylamine (2.5 g 0.025 mol) was added dropwise. The mixture was refluxed and stirred for 2–4 h. The solid product formed after cooling was removed by filtration and crystallized from ethanol. Used without analysis as it is for the next step (Figure 3) [12,13].

#### 2.2.3. Synthesis of 2-Chloro-*N*- (thiazol-2-yl) acetamide (Method C)

**Figure 4:**
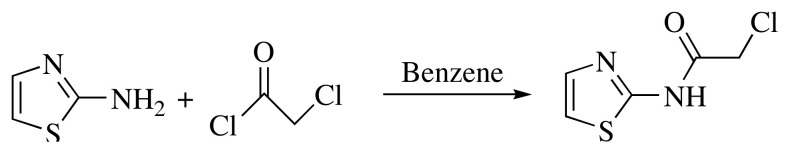
Synthesis of 2-chloro-*N*-(thiazol-2-yl) acetamide.

Chloroacetyl chloride (0.033 mol) in dry benzene (7.5 mL) was added slowly to the dry benzene 2-aminothiazole (0.02 mol) solution. The mixture was stirred at 80 °C in water bath for 3 h. Then the benzene and the excess chloroacetyl chloride were distilled off. The residue was treated with sodium bicarbonate (5% w/v) and then washed with cold water. The crude product was dried under vacuo and recrystallized from ethanol (Figure 4) [14].

#### 2.2.4. Synthesis of 2 - [(1,5-disubstituted phenyl-1*H*-imidazol-2-yl) thio] -*N*-thiazol-2-yl acetamide derivatives (Method D)

**Figure 5:**
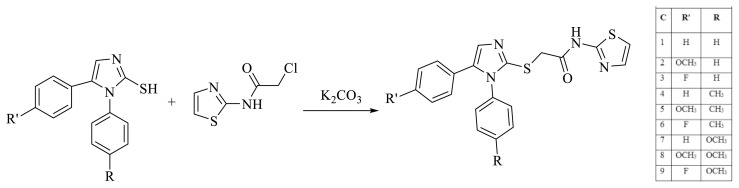
Synthesis of 2 - [(1,5-disubstituted phenyl-1*H*-imidazol-2-yl) thio] -*N*-thiazol-2-yl acetamide derivatives.

2-Mercapto-1,5-disubstituenyl-imidazole derivative (0.5 g, 0.002 mol), 2-chloro-*N*- (thiazol-2-yl) acetamide derivative and potassium carbonate solution in acetone for 5–8 h. The mixture was stirred in ice-bath. The end of the reaction was checked by TLC and the acetone remaining in the flask was evaporated. The solid product was treated with water, filtered, and then dried. The pure product is then obtained via recrystallization from ethanol (Figure 5) [13].

##### 2-((1,5-diphenyl-1*H*-imidazol-2-yl) thio) -*N*- (thiazol-2-yl) acetamide (Compound 1)

1,5-diphenyl-1*H*-imidazole-2-thiol (0.002 mol, 0.5 g), equivalent mole 2-chloro-N- (thiazol-2-yl) acetamide (0.35 g) and potassium carbonate (0.27 g) were taken in a reaction flask and synthesized according to Method D. Crude product were recrystallized from ethanol.

Yield: 73%, m.p. : 226.5 °C. IR ν_max_ (cm^−1^): 3056 (R-C=C-H), 2938 (R-CH_2_), 1682 (C=O), 1558 (C=C), 1156 (C-N). ^1^H-NMR (300 MHz, DMSO-d_6,_ ppm) δ 4.11 (2H, s, CH_2_), 7.07–7.10 (2H, m, Ar-H), 7.19–7.23 (4H, m, Ar-H), 7.29–7.32 (2H, m, thiazole C_4,5_-H), 7.34 (1H, s, imidazole C_4_-H), 7.49 (4H, d, *J*: 9, Ar-H), 12.41(1H, s, NH). ^13^C-NMR (75 MHz, DMSO-d_6,_ ppm) δ 114.1 (thiazole C_5_), 127.6, 127.8, 128.4, 128.6, 128.9, 129.6, 129.7, 130.1, 135.5, 136.2, 138.2, 143.7, 158.3 (thiazole C_2_), 167.0 (C=O). HRMS (m/z): [M+H]^+^ For C_20_H_16_N_4_OS_2_ calculated: 393.0838; found: 393.0828 (M+).

##### 2-((5-(4-methoxyphenyl)-1-phenyl-1*H*-imidazol-2-yl)thio)-*N*-(thiazol-2-yl) acetamide (Compound 2)

5-(4-methoxyphenyl)-1-phenyl-1*H*-imidazole-2-thiol (0.002 mol, 0.5 g), equimolar 2-chloro-*N*-(thiazol-2-yl) acetamide (0.31 g) and potassium carbonate (0.25 g) was taken in a reaction flask and synthesized according to Method D. The crude product was recrystallized from ethanol.

Yield: 68%, m.p.: 201.1 °C. IR ν_max_ (cm^−1^): 3075 (R-C=C-H), 2940 (R-CH_2_), 1678 (C=O), 1496 (C=C), 1250 (C-O), 1175 (C-N). ^1^H-NMR (300 MHz, DMSO-d_6,_ ppm) δ 3.69 (3H, s, OCH_3_), 4.08 (2H, s, CH_2_), 6.81 (2H, d, J: 9, 5-phenyl C_3,5_-H), 7.01( 2H, d, J: 9, 1-phenyl C_2,6_-H), 7.22–7.30 (4H, m, thiazole C_4,5_-H, Ar-H), 7.46–7.49 (4H, m, imidazol C_4_-H, Ar-H), 12.42 (1H, s, NH). ^13^C-NMR (75 MHz, DMSO-d_6,_ ppm) δ 55.6 (OCH_3_), 114.1 (thiazole C_5_), 114.4, 122.0, 127.7, 128.4, 129.2, 129.6, 130.0, 135.5, 136.2, 138.2, 142.8, 158.3, 159.1 (thiazole C_2_), 167.0 (C=O). HRMS (m/z): [M+] For C_21_H_18_N_4_O_2_S_2_ calculated: 423.0944; found: 423.0938.

##### 2-((5-(4-fluorophenyl)-1-phenyl-1*H*-imidazol-2-yl)thio)-*N*-(thiazol-2-yl) acetamide (Compound 3)

5-(4-fluorophenyl)-1-phenyl-1*H*-imidazole-2-thiol (0.002 mol, 0.5 g), equivalent mole 2-chloro-*N*- (thiazol-2-yl) acetamide (0.33 g) and potassium carbonate (0.26 g) were taken in a reaction flask and synthesized according to Method D. The crude product was recrystallized from ethanol.

Yield: 78%, m.p.: 209.6 °C. IR ν_max_ (cm^−1^): 3059 (R-C=C-H), 2908 (R-CH_2_), 1678 (C=O), 1493 (C=C), 1138 (C-N). ^1^H-NMR (300 MHz, DMSO-d_6,_ ppm) δ 4.11 (2H, s, CH_2_), 7.08–7.12 (4H, m, Ar-H), 7.22 (1H, d, *J*: 3, Ar-H), 7.29–7.32 (3H, m, thiazole C_4,5_-H, imidazol C_4_-H), 7.49 (4H, d, *J*: 9, Ar-H), 12.41 (1H, s, NH) ^13^C-NMR (75 MHz, DMSO-d_6,_ ppm) δ 114.1 (thiazole C_5_), 115.8 and 116.1 (d, CH, *^2^J*: 21.63 Hz), 126.1, 126.2, 128.4, 128.6, 129.8, 129.9, 130.1, 134.6, 136.0, 138.2, 143.6, 158.3 (thiazole C_2_), 160.2 and 163.5 (d, CF, ^1^*J*: 245.13 Hz), 167.0 (C=O). HRMS (m/z): [M+H]^+^ For C_20_H_15_FN_4_OS_2_ calculated: 411.0744; found: 411.0735.

##### 2-((5-phenyl-1-(4-methylphenyl)-1*H*-imidazol-2-yl)thio)-*N*-(thiazol-2-yl) acetamide (Compound 4)

5-phenyl-1-(4-methylphenyl)-1*H*-imidazole-2-thiol (0.002 mol, 0.5 g), equivalent mole 2-chloro-*N*- (thiazol-2-yl) acetamide (0.33 g) and potassium carbonate (0.26 g) were taken in a reaction flask and synthesized according to Method D. The crude product was recrystallized from ethanol.

Yield: 76%, m.p.: 220.8 °C. IR ν_max_ (cm^−1^): 3068 (R-C=C-H), 2939 (R-CH_2_), 1677 (C=O), 1565 (C=C), 1160 (C-N). ^1^H-NMR (300 MHz, DMSO-d_6,_ ppm) δ 2.35 (3H, s, 1-phenyl C_4_ CH_3_), 4.09 (2H, s, CH_2_), 7.10 ( 2H, d, *J*: 9, Ar-H), 7.16–7.29 (8H, m, thiazole C_4,5_-H, Ar-H), 7.32 (1H, s, imidazol C_4_-H), 7.49 (1H, d, *J*: 3, Ar-H), 12.41 (1H, s, NH). ^13^C-NMR (75 MHz, DMSO-d_6,_ ppm) δ 21.2 (CH_3_), 114.1 (thiazole C_5_), 127.6, 127.8, 128.1, 128.5, 129.0, 129.7, 130.5, 133.6, 135.5, 138.2, 139.3, 143.8, 158.3 (thiazole C_2_), 167.0 (C=O). HRMS (m/z): [M+H]^+^ for C_21_H_18_N_4_OS_2_ calculated: 407.0995; found: 407.0983.

##### 2-((5-(4-methoxyphenyl)-1-(4-methylphenyl)-1*H*-imidazol-2-yl)thio)-*N*-(thiazol-2-yl) acetamide (Compound 5)

5-(4-methoxyphenyl)-1-(4-methyl)-1H-imidazole-2-thiol (0.002 mol, 0.5 g), equimolar 2-chloro-N-(thiazol-2-yl) acetamide (0.30 g) and potassium carbonate (0.23 g) were taken in a reaction flask and synthesized according to Method D. The crude product was recrystallized from ethanol.

Yield: 70%, m.p.: 210.2 °C. IR ν_max_ (cm^−1^): 3059 (R-C=C-H), 2986 (R-CH_2_), 1672 (C=O), 1552 (C=C), 1249 (O-CH_3_), 1161 (C-N). ^1^H-NMR (300 MHz, DMSO-d_6,_ ppm) δ 2.35 (3H, s, 1-phenyl C_4_ CH_3_), 3.69 (3H, s, OCH_3_) 4.06 (2H, s, CH_2_), 6.82 ( 2H, d, *J*: 9, Ar-H), 7.03 (2H, d, *J*: 9, Ar-H), 7.16 (2H, d, *J*: 9, Ar-H), 7.21–7.23 (2H, m, thiazole C_4,5_-H), 7.25 (1H, s, Ar-H) 7.28 (1H, s, imidazol C_4_-H), 7.42 (1H, d, *J*: 6, Ar-H), 12.42 (1H, s, NH). ^13^C-NMR (75 MHz, DMSO-d_6,_ ppm) δ 21.2 (CH_3_), 55.6 (OCH_3_), 114.1 (thiazole C_5_), 114.4, 122.0, 127.6, 128.1, 129.2, 130.5, 133.6, 135.5, 138.2, 139.2, 142.9, 158.3, 159.0 (thiazole C_2_), 167.1 (C=O). HRMS (m/z): [M+H]^+^ For C_22_H_20_N_4_O_2_S_2_ calculated: 437.1100; found: 437.1092.

##### 2-((5-(4-fluorophenyl)-1-(4-methylphenyl)-1*H*-imidazol-2-yl)thio)-*N*-(thiazol-2-yl) acetamide (Compound 6)

5-(4-fluorophenyl)-1-(4-methylphenyl)-1*H*-imidazole-2-thiol (0.002 mol, 0.5 g), equimolar 2-chloro-*N*- (thiazol-2-yl) acetamide (0.31 g) and potassium carbonate (0.25 g) were taken in a reaction flask and synthesized according to Method D. The crude product was recrystallized from ethanol.

Yield: 71%, m.p.: 215.7 °C. IR ν_max_(cm^−1^): 3069 (R-C=C-H), 2990 (R-CH_2_), 1671 (C=O), 1496 (C=C), 1153 (C-N). ^1^H-NMR (300 MHz, DMSO-d_6,_ ppm) δ 2.35 (3H, s, 1-phenyl C_4_ CH_3_), 4.09 (2H, s, CH_2_), 7.09–7.16 (6H, m, Ar-H), 7.22 (1H, d, *J*: 3, Ar-H), 7.28 (2H, d, *J*: 9, thiazole C_4,5_-H), 7.30 (1H, s, imidazol C_4_-H), 7.47 (1H, d, *J*: 3, Ar-H), 12.40 (1H, s, NH). ^13^C-NMR (75 MHz, DMSO-d_6,_ ppm) δ 21.2 (CH_3_), 114.1 (thiazole C_5_), 115.8 and 116.1 (d, CH, *^2^J*: 21.68 Hz), 126.2, 128.1, 128.5, 129.8, 129.9, 130.6, 133.4, 134.6, 138.2, 139.4, 143.8, 158.3 (thiazole C_2_), 160.2 and 163.4 (d, CF, ^1^*J*: 245.04 Hz), 167.0 (C=O). HRMS (m/z): [M+H]^+^ For C_21_H_17_FN_4_OS_2_ calculated: 425.0901; found: 425.0890.

##### 2-((1-(4-methoxyphenyl)-5-phenyl-1*H*-imidazol-2-yl)thio)-*N*-(thiazol-2-yl) acetamide (Compound 7)

1-(4-methoxyphenyl)-5-phenyl-1*H*-imidazole-2-thiol (0.002 mol, 0.5 g), equimolar 2-chloro-*N*- (thiazol-2-yl) acetamide (0.31 g) and potassium carbonate (0.25 g) were taken in a reaction flask and synthesized according to Method D. The crude product was recrystallized from ethanol.

Yield: 73%, m.p.: 207.2 °C. IR ν_max_ (cm^−1^): 3059 (R-C=C-H), 2939 (R-CH_2_), 1672 (C=O), 1560 (C=C), 1253 (O-CH_3_), 1162 (C-N). ^1^H-NMR (300 MHz, DMSO-d_6,_ ppm) δ 3.79 (3H, s, OCH_3_), 4.09 (2H, s, CH_2_), 7.01 (2H, d, *J*: 9, Ar-H), 7.09–7.13 (2H, m, Ar-H), 7.20–7.27 (6H, m, thiazole C_4,5_-H, Ar-H), 7.32 (1H, s, imidazol C_4_-H), 7.48 (1H, d, *J*: 6, Ar-H), 12.42 (1H, s, NH). ^13^C-NMR (75 MHz, DMSO-d_6,_ ppm) δ 55.9 (OCH_3_), 114.1 (thiazole C_5_), 115.1, 127.5, 127.8, 128.4, 128.7, 129.0, 129.6, 129.7, 135.6, 138.2, 144.1, 158.3, 159.9, 166.9 (thiazole C_2_), 167.0 (C=O). HRMS (m/z): [M+H]^+^ For C_21_H_18_N_4_O_2_S_2_ calculated: 423.0944; found 423.0938.

##### 2-((1,5-bis(4-methoxyphenyl)-1*H*-imidazol-2-yl)thio)-*N*-(thiazol-2-yl) acetamide (Compound 8)

1,5-bis (4-methoxyphenyl)-1*H*-imidazole-2-thiol (0.002 mol, 0.5 g), equivalent mole 2-chloro-*N*- (thiazol-2-yl) acetamide (0.28 g) and potassium carbonate (0.22 g) were taken in a reaction flask and synthesized according to Method D. The crude product was recrystallized from ethanol.

Yield: 70%, m.p.: 206.9 °C. IR ν_maks_ (cm^−1^): 3068 (R-C=C-H), 2970 (R-CH_2_), 1670 (C=O), 1550 (C=C), 1254 (O-CH_3_), 1142 (C-N). ^1^H-NMR (300 MHz, DMSO-d_6,_ ppm) δ 3.70 (3H, s, OCH_3_), 3.79 (3H, s, OCH3), 4.05 (2H, s, CH_2_), 6.82 ( 2H, d, *J*: 9, Ar-H), 7.02 (4H, dd, *J*: 6, Ar-H), 7.20–7.23 (4H, m, tiyazol C_4,5_-H, imidazol C_4_-H, Ar-H), 7.48 (1H, d, *J*: 3, Ar-H), 12.42 (1H, s, NH). ^13^C-NMR (75 MHz, DMSO-d_6,_ ppm) δ 55.55 (OCH_3_), 55.9 (OCH_3_), 114.1 (thiazole C_5_), 114.4, 115.1, 122.1, 127.5, 128.7, 129.1, 129.7, 135.6, 138.2, 159.0, 159.9 (thiazole C_2_), 167.1 (C=O). HRMS (m/z): [M+H]^+^ For C_22_H_20_N_4_O_3_S_2_ calculated: 453.1050; found 453.1039.

##### 2-((5-(4-fluorophenyl)-1-(4-methoxyphenyl)-1*H*-imidazol-2-yl)thio)-*N*-(thiazol-2-yl) acetamide (Compound 9)

5-(4-fluorophenyl)-1-(4-methoxyphenyl)-1*H*-imidazole-2-thiol (0.002 mol, 0.5 g), equivalent molar 2-chloro-*N*-(thiazol-2-yl) acetamide (0.29 g) and potassium carbonate (0.23 g) were taken in a reaction flask and synthesized according to Method D.

Yield: 75%, m.p.: 200.1 °C. IR ν_max_ (cm^−1^): 3053 (R-C=C-H), 2938 (R-CH_2_), 1652 (C=O), 1512 (C=C), 1221 (O-CH_3_), 1147 (C-N). ^1^H-NMR (300 MHz, DMSO-d_6,_ ppm) δ 3.79 (3H, s, OCH_3_), 4.09 (2H, s, CH_2_), 7.00 (2H, d, *J*: 9, Ar-H), 7.10–7.18 (4H, m, Ar-H), 7.22–7.25 (3H, m, thiazole C_4,5_-H, Ar-H), 7.31 (1H, s, imidazol C_4_-H), 7.48 (1H, d, *J*: 3, Ar-H), 12.42 (1H, s, NH). ^13^C-NMR (75 MHz, DMSO-d_6,_ ppm) δ 55.9 (OCH_3_), 114.1 (thiazole C_5_), 115.2, 115.8 and 116.1 (d, CH, *^2^J*: 21.62 Hz), 126.2, 126.3, 128.3, 128.5, 128.7, 129.7, 129.7, 129.8, 134.7, 138.2, 144.01, 158.30, 160.0 (thiazole C_2_), 160.2 and 163.4 (d, CF, ^1^*J*: 245.10 Hz), 167.0 (C=O). HRMS (m/z): [M+H]^+^ For C_21_H_17_FN_4_O_2_S_2_ calculated: 441.0850; found: 441.0845.

### 2.3. COX-1 and COX-2 enzyme activity

All of the synthesized compounds were tested for their bot COX-1 and COX-2 inhibition activity. The inhibition assay was started by dissolving the products in DMSO. In order to analyze the actual enzyme inhibition of the test molecules, the effect of DMSO was removed and the DMSO in the solutions was kept below 1%. Enzyme inhibition experiment (COX Fluorescent Inhibitor Screening Assay Kit, Cayman Chemical Company, Ann Arbor, MI, USA 700100) was carried out in line with the manufacturer’s instructions and the enzyme inhibition of the compounds was performed in a multimode microplate reader with an excitation wavelength of 530–540 nm and an emission wave of 585–595 nm measured by length. The enzyme inhibition of the compounds at a concentration of 10 μM was calculated by comparing according to the standards included in the kit (SC-560 for COX-1, DuP-697 for COX-2) [15].

The calculation was performed by the manufacturer’s instructions;


%Inhibition=[(Initial activity-Sample activity)/Initial Activity]×100

### 2.4. Molecular modeling studies

During molecular modeling studies, crystallographic data of “Cyclooxygenase 2 structure complex with a selective inhibitor SC-558” (Pdb ID; 1CX2) and “Crystal Structure of Cyclooxygenase-1 in complex with celecoxib” (Pdb ID; 3KK6) were downloaded from Research Collaboratory for Structural Bioinformatics (RCSB) protein database and used in pdb format [16–18].

Protein structure preparation, GRID files used in docking operations, docking/scoring with various algorithms and visualization were carried out with Maestro (Schrodinger Inc, USA) software and related subunits. All processes requiring a computer were carried out using workstations within the School of Pharmacy of Istanbul Medipol University.

#### 2.4.1. Preparation of the ligand set to be used

The structure of all the components has been drawn in three dimensions with the help of Maestro (Schrodinger Inc, USA) program modules. The energies of the structures were minimized by using the ligprep module, their pH 7 (+/− 2) ionized forms and tautomers were prepared, repeating and salt form ones were removed.

#### 2.4.2. Preparation of the target to be used

Crystallographic data of “cyclooxygenase 2 structure complexes with a selective inhibitor SC-558” (Pdb ID; 1CX2) and “Cyclooxygenase 1 crystal structure in complex with Celecoxib” (Pdb ID; 3KK6) were downloaded in PDB format. [19,20]. Subsequently, the hydrogens of the structure were added with the help of the “protein preparation wizard” under Maestro (Schrodinger Inc, USA) program and the bond orders were rearranged in accordance with the software. After determining the appropriate side chain positioning of amino acids and possible intraprotein H bonds, the hydrogens of the entire structure were minimized using Optimized Potentials for Liquid Stimulations (OPLS) 2005 energy parameters.

#### 2.4.3. Preparation of GRIDs belonging to the active region

Using the Glide-Grid preparation module of Maestro (Schrodinger Inc, USA), various interaction maps of the active region were prepared. During the preparation of the file, the original ligands were taken as the center point, and Van der Waals radius scaling factors were kept as default. No other restrictions were added.

#### 2.4.4. Docking and scoring

With the help of optimized GRID files, first original ligands (for the purpose of internal validation) then previously prepared ligands were docked by HTVS and XP protocols of Glide-docking interface (Maestro, Schrodinger Inc). The poses of original ligands and compound with highest biological activities were visually inspected. Docking/maximum e-model scores of XP protocol were recorded.

#### 2.4.5. Preparation of interaction charts and figures

The graphics and interactions resulting from the calculations were saved in high resolution picture format and placed in the text content with the help of Maestro (Schrodinger Inc) software 2D/3D visualization tools.

#### 2.4.6. Molecular predictions

Maestro (Schrodinger Inc.) QikProp module was used to predict drug-likeness properties of synthesized compounds. CNS activity, logPo/w predicted octanol/water coefficient), HERG K+ channel inhibition IC_50_ value, logBB (blood/brain partition) logKHSA: (human serum albumin binding) and Human Oral Absorption (HOA), Percent HOA were chosen as main predictors.

## 3. Results

### 3.1. Chemistry

In this study, nine original 2 - [(1,5-disubstituentiphenyl-1*H*-imidazol-2-yl) thio] -*N*- (2-thiazolyl) acetamide derivatives were synthesized at 68%–78% yield. IR values of all compounds for 2-[(1,5-disubstitutedphenyl-1*H*-imidazol-2-yl)thio]-*N*-(2-thiazolyl) acetamide derivatives, C=O stretching bands were observed between 1671–1682 cm^−1^. Aliphatic C-H stretching bands were observed between 2902 and 2990 cm^−1^ and R-C=C-H bands were observed between 3053 and 3075 cm^−1^. C=C stretching bands for all acetamide derivatives were observed between 1565 and 1494 cm^−1^ as expected. The C-N stretching band was observed between 1130 and 1175 cm^−1^ as a moderate band. The C-O stretching bands for the compounds containing methoxy group (compound 2, 5, 7, 8, and 9) was observed around 1250 cm^−1^. No specific band was observed due to the presence of more than one aromatic ring in the fingerprint region.

The methylene (-CH_2_-) group adjacent to the carbonyl and sulfur underwent chemical shift as expected in all compounds, giving 2H and singlet peak in the range 4.05–4.11 ppm. NH hydrogen in amide structure found in all our structures gave a singlet and flat peak in the range of 12.40–12.42 ppm. The molecules consist of 11–13 protons in total belonging to their aromatic rings (thiazole, imidazole, and phenyl). All the protons belonging to aromatic structures were observed in all spectrums. The only hydrogen in the imidazole ring was observed as a separate singlet in compounds **1**, **2**, **4**, **5**, **6**, **7**, and **9**, with the other compounds (**3**, **8**) being overlapped with thiazole and/or phenyl aromatic hydrogens. 2 hydrogen on the thiazole were observed as 2H and doublet in compounds **1**, **5**, and **6**. It is overlapped with other aromatic hydrogens in other compounds. The aromatic methyl group containing compounds **4**, **5**, and **6** were observed with 2.35 ppm as 3H values. In the compounds **2**, **5**, **7**, **8**, and **9** containing methoxyphenyl groups, the methyl group underwent chemical shift and was observed between 3.69 and 3.79 ppm. Methoxy groups on the phenyl at 1st position of imidazole were observed at 3.79 ppm, and the ones on the phenyl at fifth position of imidazole were observed at 3.69 ppm.

### 3.2. COX-1 and COX-2 enzyme activity results

**Figure 6:**
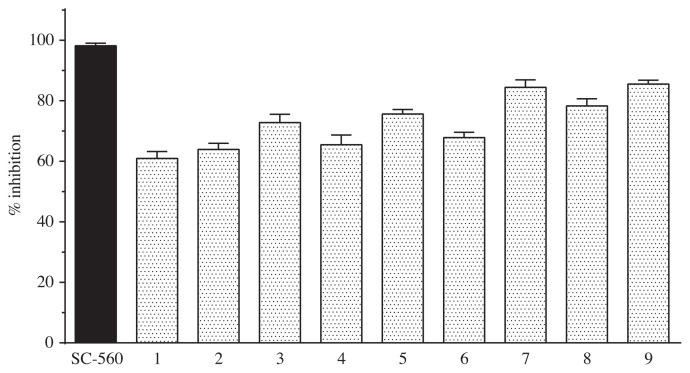
COX-1 enzyme inhibition analysis of SC-560 and nine compounds.

**Figure 7:**
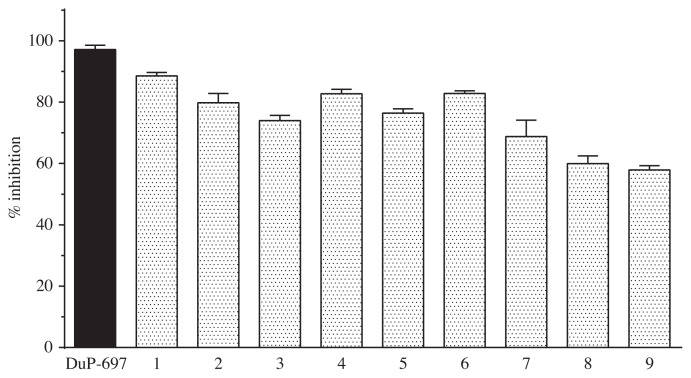
COX-2 enzyme inhibition analysis of DuP-697 and nine compounds.

In order to test the COX-1 and COX-2 enzyme inhibition effects of the compounds, procedures were carried out as recommended by the manufacturer. For this purpose, enzymes and compounds were left to incubation, then 200 μL of enzyme containing tested compounds was put into wells at a final concentration of 10 μM. The enzyme activity for each compound was carried out triplicate. One-way ANOVA (one-way ANOVA) test was applied to analyze the consistency between replicates of the experimental groups. The significance of the groups compared to the standard inhibitors (SC-560 for COX-1, DuP-697 for COX-2) group was evaluated by Dunnett’s test. COX-1 and COX-2 enzyme inhibition in 10 μM concentration of SC-560 and DuP-697 used as standard inhibitory compounds. Standards and nine compounds enzyme activity results are given in Figures 6 and 7 and Table 1.

The effects of compounds and SC-560 (98.2% inhibition) used as standard inhibitor on COX-1 enzyme activity were determined. Compound **9** and compound **7** were found to be the most active on COX-1 enzyme with 85.5 % and 82.4 % inhibition respectively. Compound **2** showed 63% and compound **4** showed approximately 65% inhibitory activity on COX-1. The obtained activity values were statistically significant (at least p < 0.05).

The effects of the synthesized compounds and DuP-697 (97.2% inhibition) used as standard inhibitor on COX-2 enzyme activity were determined. Among the tested compounds, compound 1, compound 6, and compound 4 were found to be the most active compounds on COX-2 enzyme with 88.5%, 82.8%, and 82.7% inhibition, respectively. The least active compound was found to have 57% inhibition value among all of the tested products. The obtained activity values were statistically significant (at least p < 0.05).

### 3.3. Molecular modeling

For validation of docking studies, original ligands SC-558 and Celecoxib were successfully redocked to their crystallographic data by the same protocols used for designed ligands without any restrictions. The RMSD values of these ligands compared to their original positions are 1.1097 and 0.3910, respectively.

#### 3.3.1. COX-2 molecular interactions

**Figure 8:**
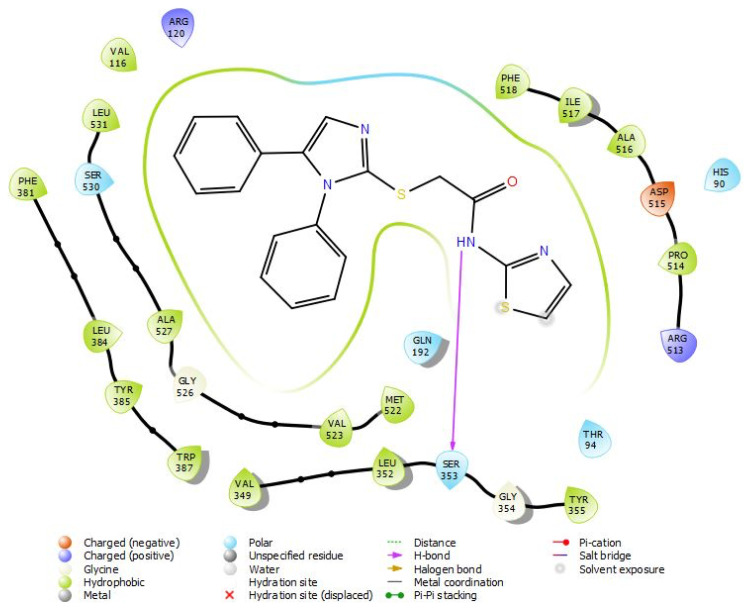
Two-dimensional interaction map of compound **1**-COX-2 protein structure.

**Figure 9:**
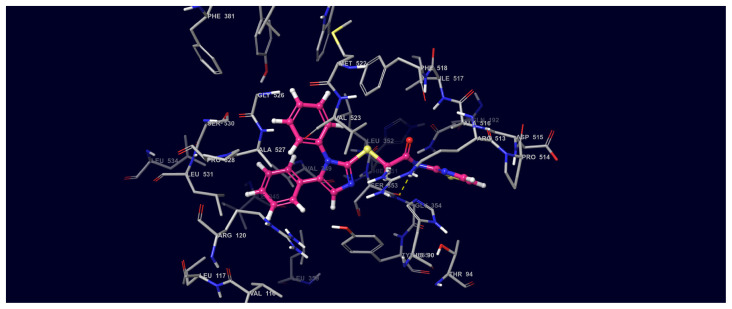
Three-dimensional interaction map of compound **1**-COX-2 protein structure (Compound 1 C: pink, N: blue, S: yellow, O: red).

The 2D and 3D interaction potentials of the docked Compound 1-COX-2 enzyme were analyzed. Interactions between ring nitrogen, carbonyl, phenyl, secondary amine functional groups and active site amino acids are detailed in Figures 8 and 9.

In the compound **1**; the phenyl group at 5th position of imidazole interacts with the Arg120 amino acid via hydrophobic effects in the active site. The 4th carbon atom attached to the same phenyl ring (at 5th position of imidazole) is in hydrophobic interaction with Leu 531, while the carbons of same phenyl (number 2, 3, and 5) are in hydrophobic interaction with Val 349. The amino acids Ala 516 and Thr 94 in the active site hydrophobically interact with the 5th carbon of the thiazole ring in its main structure.

Phenyl group on the first position of imidazole establishes hydrophobic interactions with the Trp 387 and Val 523 on COX-2 active site. The N atom of the acetamide moiety in the structure of compound **1** forms hydrogen bond with the Trp 387 and Ser 353 amino acids.

#### 3.3.2. COX-1 molecular interactions

**Figure 10:**
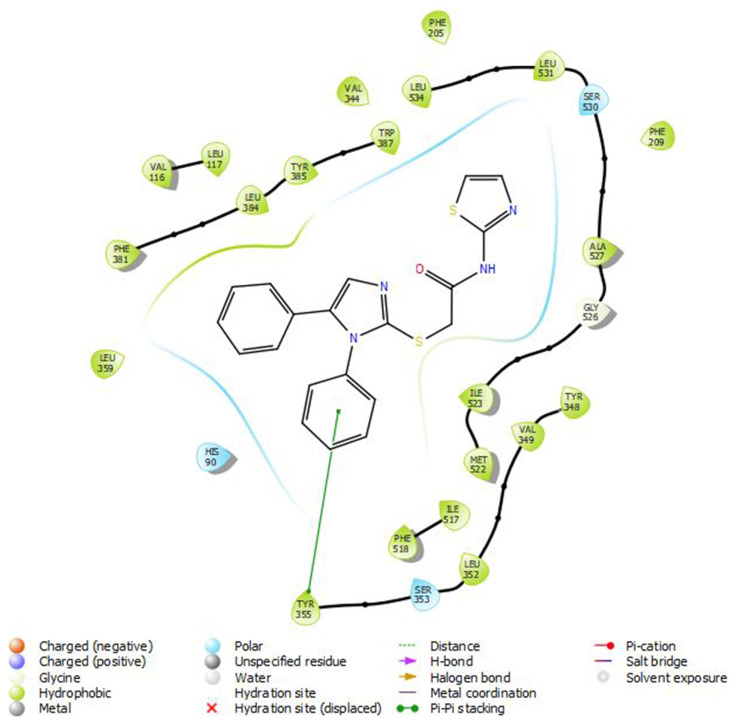
Two-dimensional interaction map of compound **1**-COX-1 protein structure.

**Figure 11:**
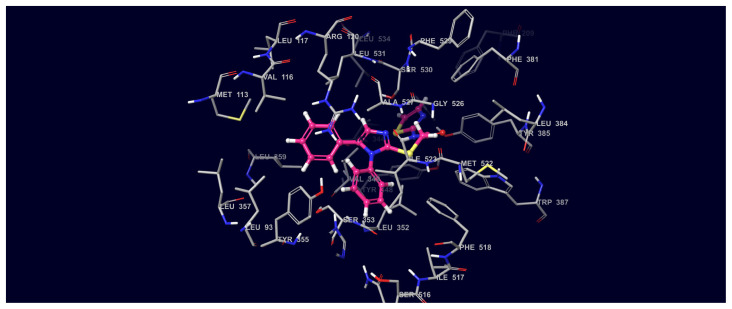
Three-dimensional interaction map of compound **1**-COX-1 protein structure (Compound 1 C: pink, N: blue, S: yellow, O: red).

The 2D and 3D interaction potentials of the docked Compound 1-COX-1 enzyme were analyzed in Figures 10 and 11. Interactions between ring nitrogen, carbonyl, phenyl, secondary amine functional groups and active site amino acids are detailed.

Phenyl ring in the 5th position of the imidazole form hydrophobic interactions with Val116, Leu115, Leu531, Leu359 and Tyr355 in the COX-1 active site. The Val344 interacts hydrophobically with the S atom of the thiazole ring in the compound **1**. Phenyl ring on the first position of imidazole forms hydrophobic interaction with Ile 517 and Tyr355 (Figure 11).

Docking/maximum e-model scores of orginal ligands and compound 1 are also calculated during the docking process. (Table 2)

Calculated drug-likeness properties of the synthesized compounds were tabulated in Table 3.

## 4. Discussion

All of the structure characterizations of the synthesized compounds were performed with spectroscopic methods. ^1^H NMR, ^13^C NMR, and MS spectra resulted in expected chemical structures. For compounds **3**, **6**, and **9**, the fluorine couplings were determined. Aromatic fluorine splits were observed around 240–250 Hz for the aromatic carbon directly bound to F, and 20–25 Hz for first neighborhood C-H carbons of the phenyl. Accordingly, C-F carbon splitting (160 and 163 ppm, *J:* 245 Hz) is consistent with the literature data. C-H carbon splittings in the neighborhood of fluorine (115.8 and 116.02, *J:* 21.6 Hz) also met the literature and expected data [21].

The COX-1 and COX-2 inhibition activities of the compounds are studied against the SC-560 and DuP-697 standards, respectively. Enzyme-active site interactions of the active compound were investigated with the support of molecular modeling studies which were internally validated previously with satisfactory RMSD findings. Compound **1** has selective COX-2 enzyme activity; however, COX-1 inhibition was found to be rather low (60.9%) compared to standard SC-560 (98.2% inhibition). When examined in terms of selective activity, the binding of methoxy and fluorine functionalities to the p- position of the phenyl ring in the 5th position to the imidazole ring, partially favors the selective COX-2 inhibition. Even if the scoring functions do not indicate an expected activity in general, particularly solvation parameters included glide e-model scores showed parallel relations with the activity results.

The -NH- group of the acetamide in the structure of all compounds makes the H-bond with Ser 353 of COX-2. This situation does not occur in the COX-1 structure. The difference in the binding sites between the two enzymes is not the separation of amino acid sequences, but rather the volumetric differences. This situation manifests itself as the difference in size although there are hydrophobic pockets in both structures. The substituents in the phenyl rings on the structure of the compounds are important in terms of selectivity, rather than showing the H-bond acceptor or donor feature, they fit into these pockets by volume. The most active compounds for COX-2 are 1, 4, 6, and 7, those having H or F on the phenyl ring attached at the 5th position of imidazole. Also, the compound with the lowest inhibition is compound **9** on COX-2. Selective inhibition of COX-2 can be reduced by electron withdrawing groups located on phenyl ring. In the COX-1inhibitory activity, compounds **7–9** was found to be the most active ones, including methoxy group on the phenyl attached at 1st position of imidazole.

In this study, COX inhibition levels were measured by a single concentration. Thus, it is not possible to directly correlate with similar studies. However, at 10 μM concentration, inhibition rate is up to 50% for COX-1 and up to 70% for COX-2. Selective COX-2 inhibitors usually show their effect at submicromolar level. Similar compounds with trisubstituted five-membered cycles have been synthesized before, and the IC_50_ values are in a range of 3–50 μM concentration, where the IC_50_ value of celecoxib was found to be 0.30 μM [22–25]. The existence of electron-withdrawing substituents attached to the ring system reduces the hydrophobicity of systems that fit into hydrophobic pockets, and the degree of bonding decreases due to pi-pi interactions. This situation is reflected in the activity as nonselectivity.

As a result of these studies, it was determined that the compounds bearing substituted imidazolyl acetamide derivative showed selective COX-2 inhibition effect within limits calculated drug-likeness properties. In future studies, it is planned to work with different concentrations, to try ring systems that can show similar properties, as well as to obtain more effective compounds by increasing the variety of substituents, as well as to examine the kinetic properties of these compounds.

## SUPPLEMENTARY MATERIALS

Figure S.1 ^1^H-NMR spectrum of Compound 1

Figure S.2 ^1^H-NMR spectrum of Compound 2

Figure S.3 ^1^H-NMR spectrum of Compound 3

Figure S.4 ^1^H-NMR spectrum of Compound 4

Figure S.5 ^1^H-NMR spectrum of Compound 5

Figure S.6 ^1^H-NMR spectrum of Compound 6

Figure S.7 ^1^H-NMR spectrum of Compound 7

Figure S.8 ^1^H-NMR spectrum of Compound 8

Figure S.9 ^1^H-NMR spectrum of Compound 9

Figure S.10 IR spectrum of Compound 1

Figure S.11 IR spectrum of Compound 2

Figure S.12 IR spectrum of Compound 3

Figure S.13 IR spectrum of Compound 4

Figure S.14 IR spectrum of Compound 5

Figure S.15 IR spectrum of Compound 6

Figure S.16 IR spectrum of Compound 7

Figure S.17 IR spectrum of Compound 8

Figure S.18 IR spectrum of Compound 9

Figure S.19 ^13^C NMR spectrum of Compound 1

Figure S.20 ^13^C NMR spectrum of Compound 2

Figure S.21 ^13^C NMR spectrum of Compound 3

Figure S.22 ^13^C NMR spectrum of Compound 4

Figure S.23 ^13^C NMR spectrum of Compound 5

Figure S.24 ^13^C NMR spectrum of Compound 6

Figure S.25 ^13^C NMR spectrum of Compound 7

Figure S.26 ^13^C NMR spectrum of Compound 8

Figure S.27 ^13^C NMR spectrum of Compound 9

Figure S.28 HRMS spectrum of Compound 1

Figure S.29 HRMS spectrum of Compound 2

Figure S.30 HRMS spectrum of Compound 3

Figure S.31 HRMS spectrum of Compound 4

Figure S.32 HRMS spectrum of Compound 5

Figure S.33 HRMS spectrum of Compound 6

Figure S.34 HRMS spectrum of Compound 7

Figure S.35 HRMS spectrum of Compound 8

Figure S.36 HRMS spectrum of Compound 9

## Figures and Tables

**Table 1:** Percent enzyme inhibition values ± SD of SC-560 and DuP-697 used as standard inhibitors of the synthesized compounds.

Compound	COX-1	COX-2
1	60.9 ± 2.31	88.5 ± 1.12
2	63.9 ± 2.03	79.8 ± 3.06
3	72.8 ± 2.68	76.8 ± 3.17
4	65.5 ± 3.18	82.7 ± 1.43
5	75.6 ± 1.48	76.4 ± 1.41
6	67.8 ± 1.78	82.8 ± 0.89
7	82.4 ± 1.81	64.0 ± 3.01
8	78.3 ± 2.35	60.0 ± 2.52
9	85.5 ± 1.30	57.9 ± 1.39
SC-560	98.2 ± 0.88	-
DuP-697	-	97.2 ± 1.36

**Table 2:** Docking and Glide e-model scores of original ligands and compound 1 calculated from XP docking protocol.

Compound	Pdb id	Docking Score	Glide e–model score
SC–558	1CX2	−8.984	−74.195
1	1CX2	−2.348	−45.863
Celecoxib	3KK6	−12,448	−80.230
1	3KK6	−8.533	−27.507

**Table 3:** Drug-likeness properties of compounds.

**C** ^*^	CNS	log_Po/w_	log_HERG_	P_Caco_	logBB	PMDCK	logKHSA	HOA	%HOA
**1**	0	4.79	−7.40	1411.88	−0.45	1931.70	0.63	1	100.00
**2**	0	4.43	−5.60	2152.07	−0.19	2963.54	0.45	3	100.00
**3**	0	5.04	−7.29	1402.27	−0.35	3460.41	0.68	1	100.00
**4**	0	5.10	−7.29	1411.89	−0.48	1931.12	0.79	1	100.00
**5**	0	4.64	−5.39	2214.88	−0.18	2987.23	0.56	3	100.00
**6**	0	5.34	−7.16	1411.83	−0.37	3486.11	0.83	1	100.00
**7**	0	4.34	−5.38	2272.41	−0.16	2959.04	0.41	3	100.00
**8**	0	4.98	−7.15	1435.67	−0.61	1977.45	0.64	1	100.00
**9**	0	4.58	−5.29	2284.62	−0.05	5389.59	0.46	3	100.00

Descriptor: Predicted central nervous system (CNS) activity on a scale of −2 (inactive) to +2 (active). logPo/w: Predicted octanol/water partition coefficient (−2.0 to 6.5). log_HERG_: Predicted IC_50_ value for blockage of HERG K^+^ channels (below −5) logBB: Predicted brain/blood partition coefficient (−3.0 to 1.2). logK_HSA_: Prediction of binding to human serum albumin (−1.5 to 1.5). Human Oral Absorption (HOA): Predicted qualitative human oral absorption measured as 1, 2, or 3 for low. Percent HOA: high if >80% and poor if <25%.
