# Supplementary figures and images for: Synthesis, characterization, COX1/2 inhibition and molecular modeling studies on novel 2-thio-diarylimidazoles
Source: Turk J Chem. 2021 Aug 6;45(6):1841–53. doi: 10.3906/kim-2104-54 (PMC10734748; doi:10.3906/kim-2104-54)

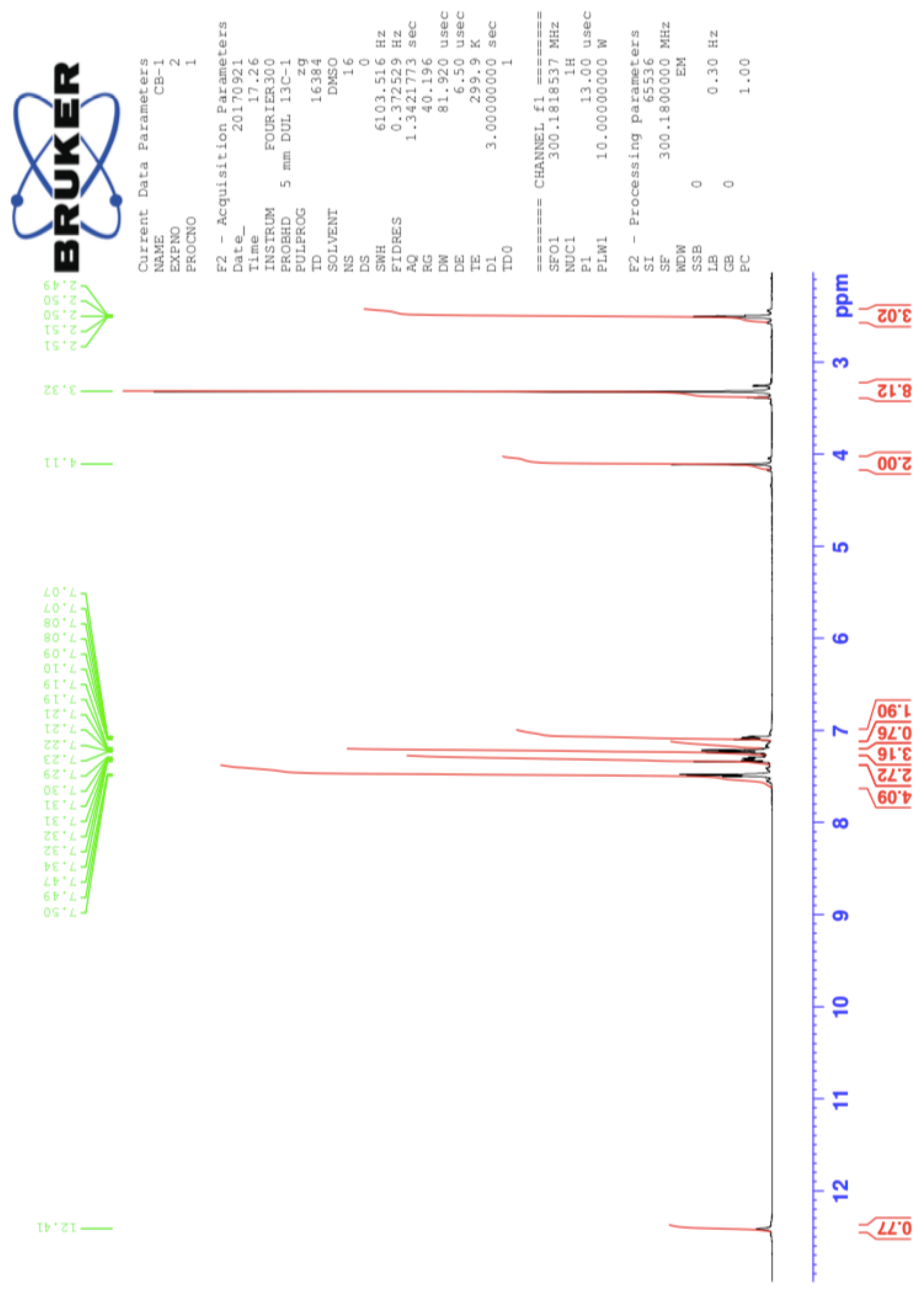

Supplement: Figure S.1 — 1H-NMR spectrum of Compound 1 [file turkjchem-45-6-1841s1.tif]

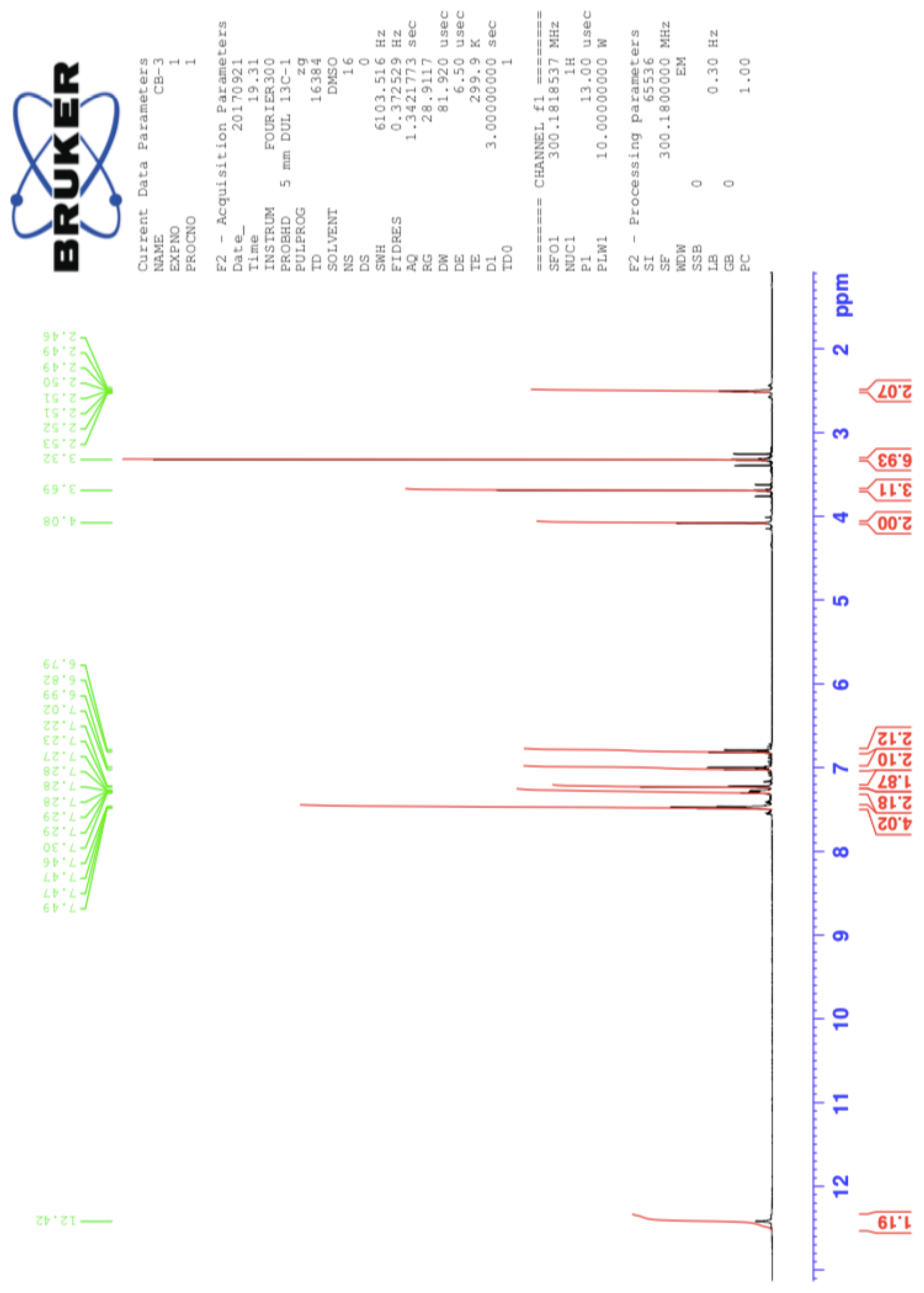

Supplement: Figure S.2 — 1H-NMR spectrum of Compound 2 [file turkjchem-45-6-1841s2.tif]

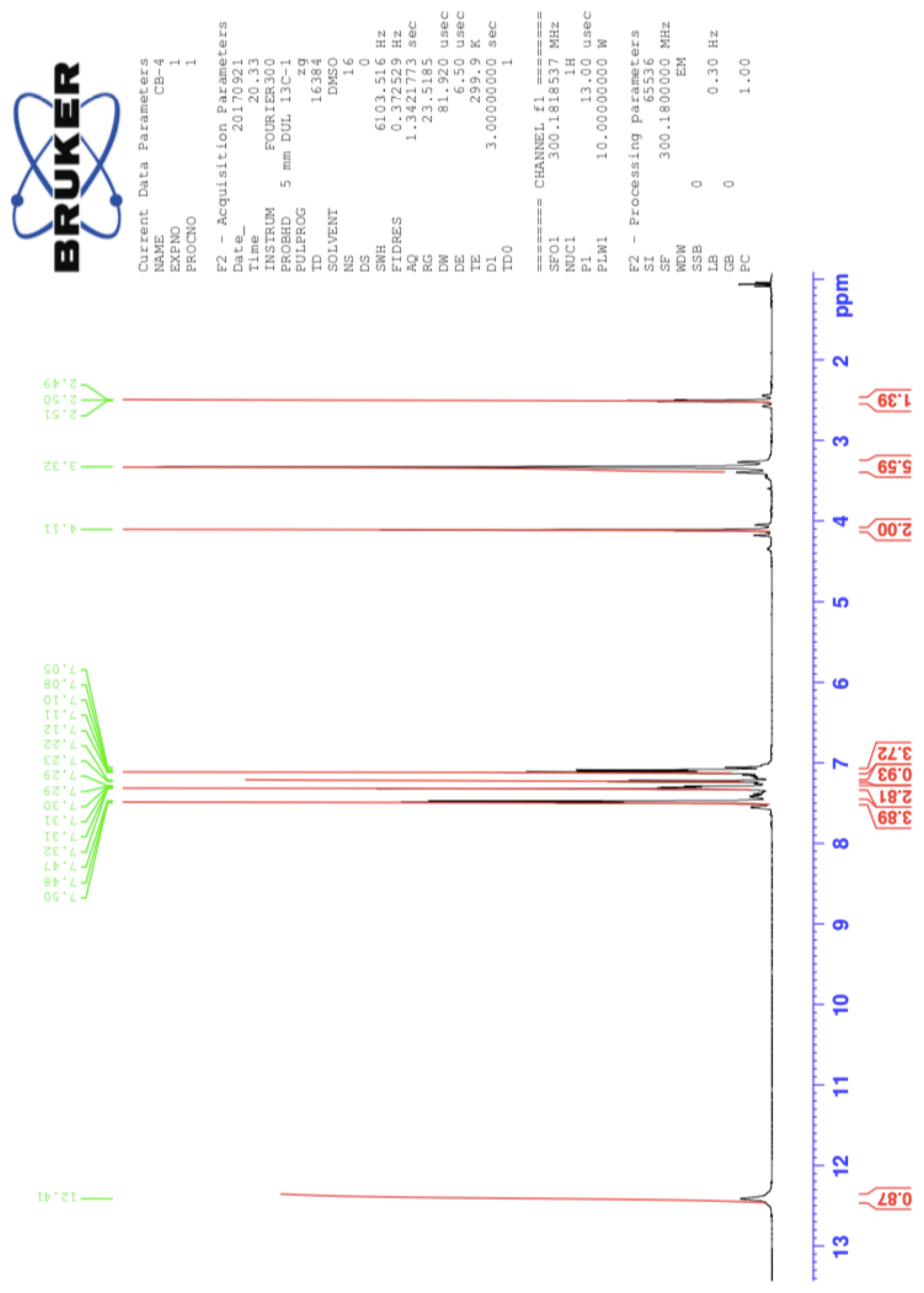

Supplement: Figure S.3 — 1H-NMR spectrum of Compound 3 [file turkjchem-45-6-1841s3.tif]

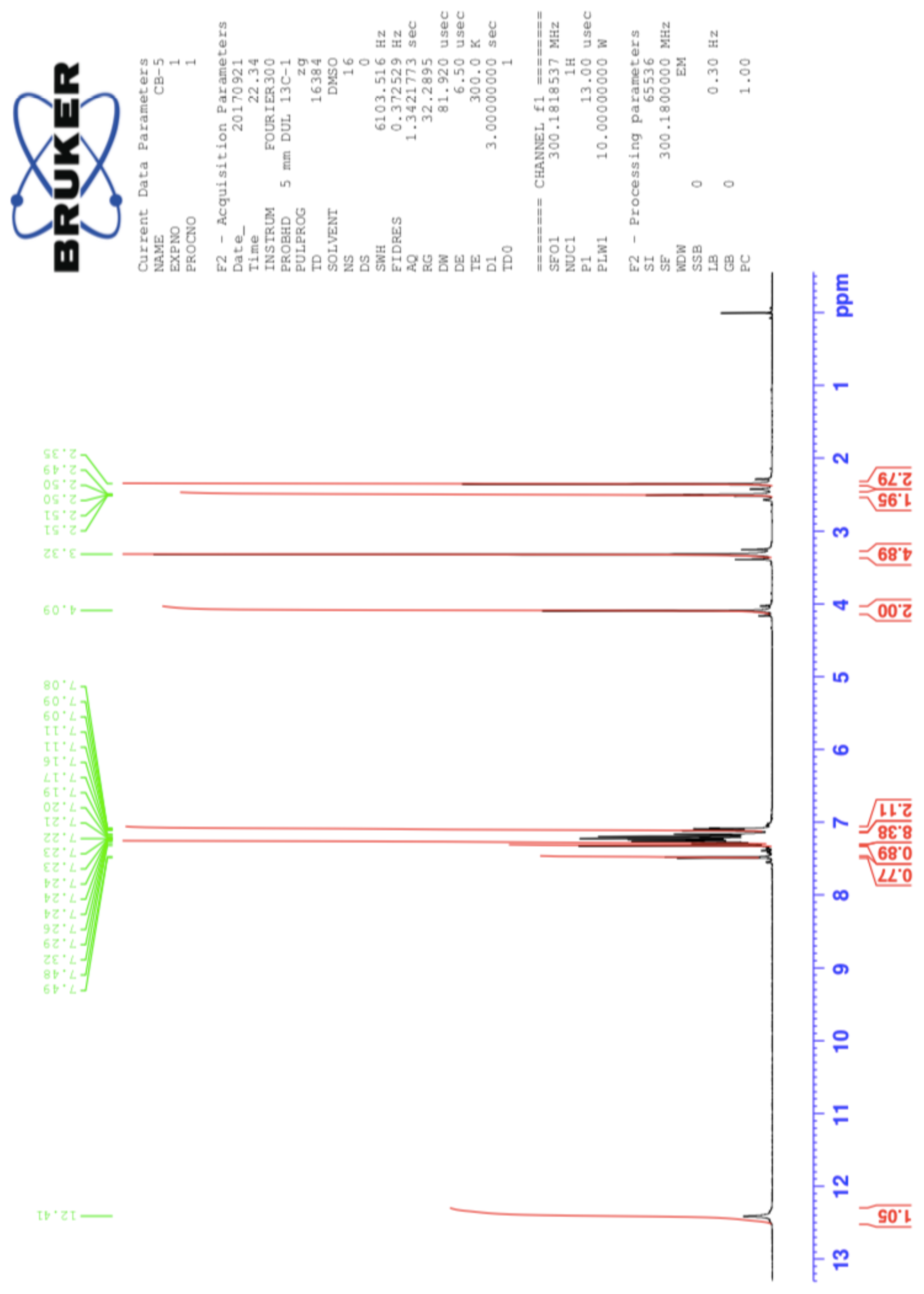

Supplement: Figure S.4 — 1H-NMR spectrum of Compound 4 [file turkjchem-45-6-1841s4.tif]

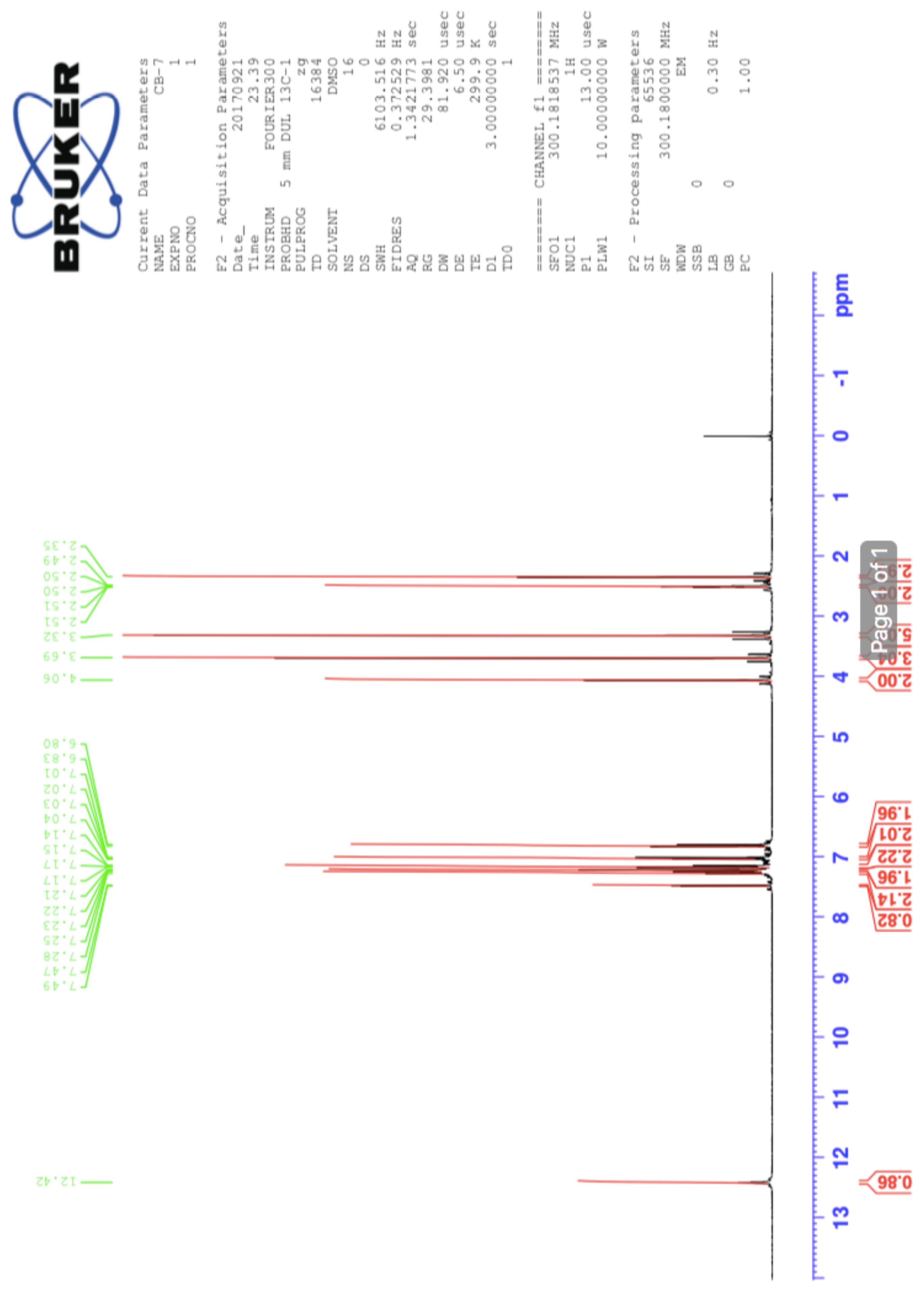

Supplement: Figure S.5 — 1H-NMR spectrum of Compound 5 [file turkjchem-45-6-1841s5.tif]

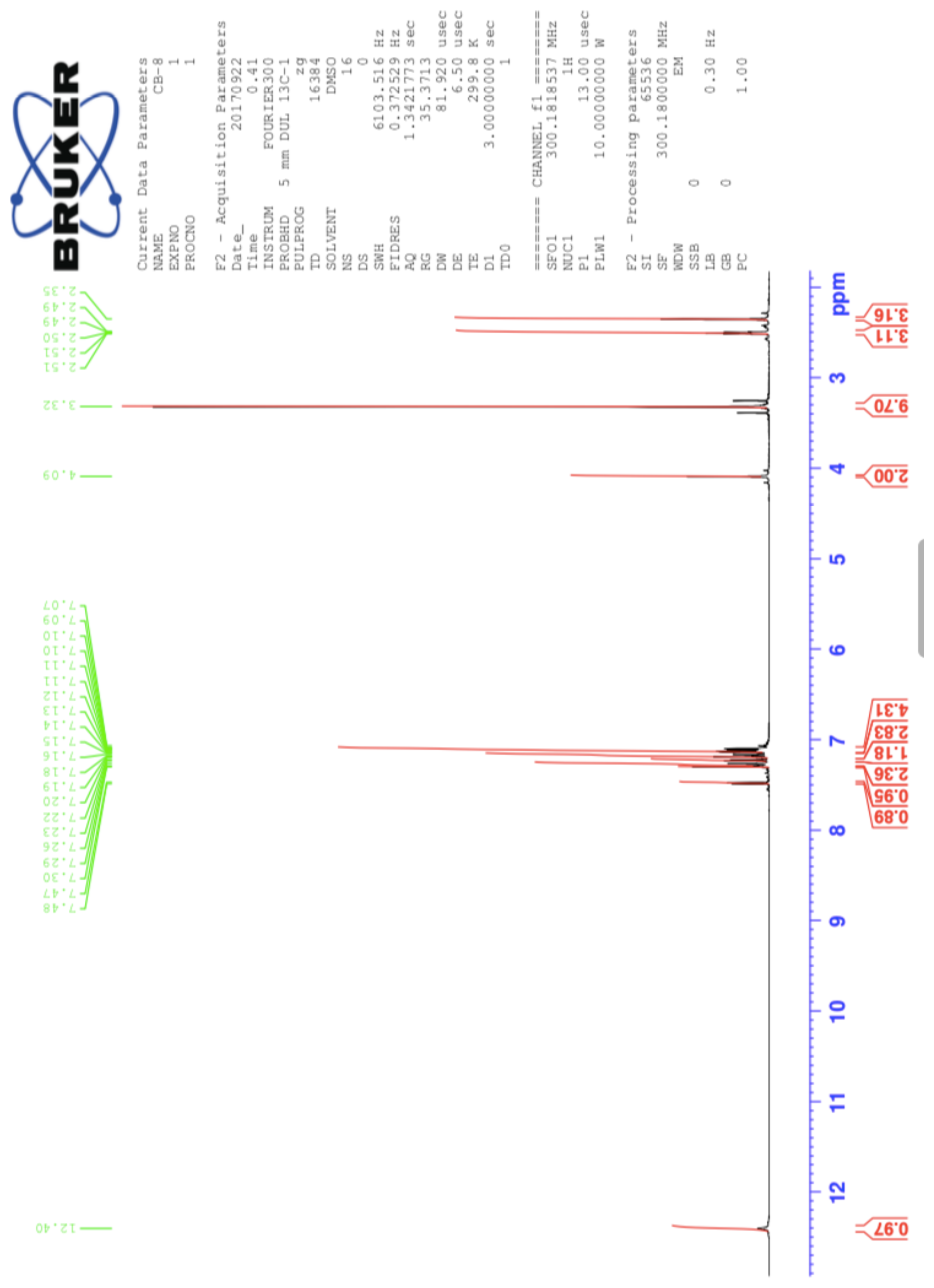

Supplement: Figure S.6 — 1H-NMR spectrum of Compound 6 [file turkjchem-45-6-1841s6.tif]

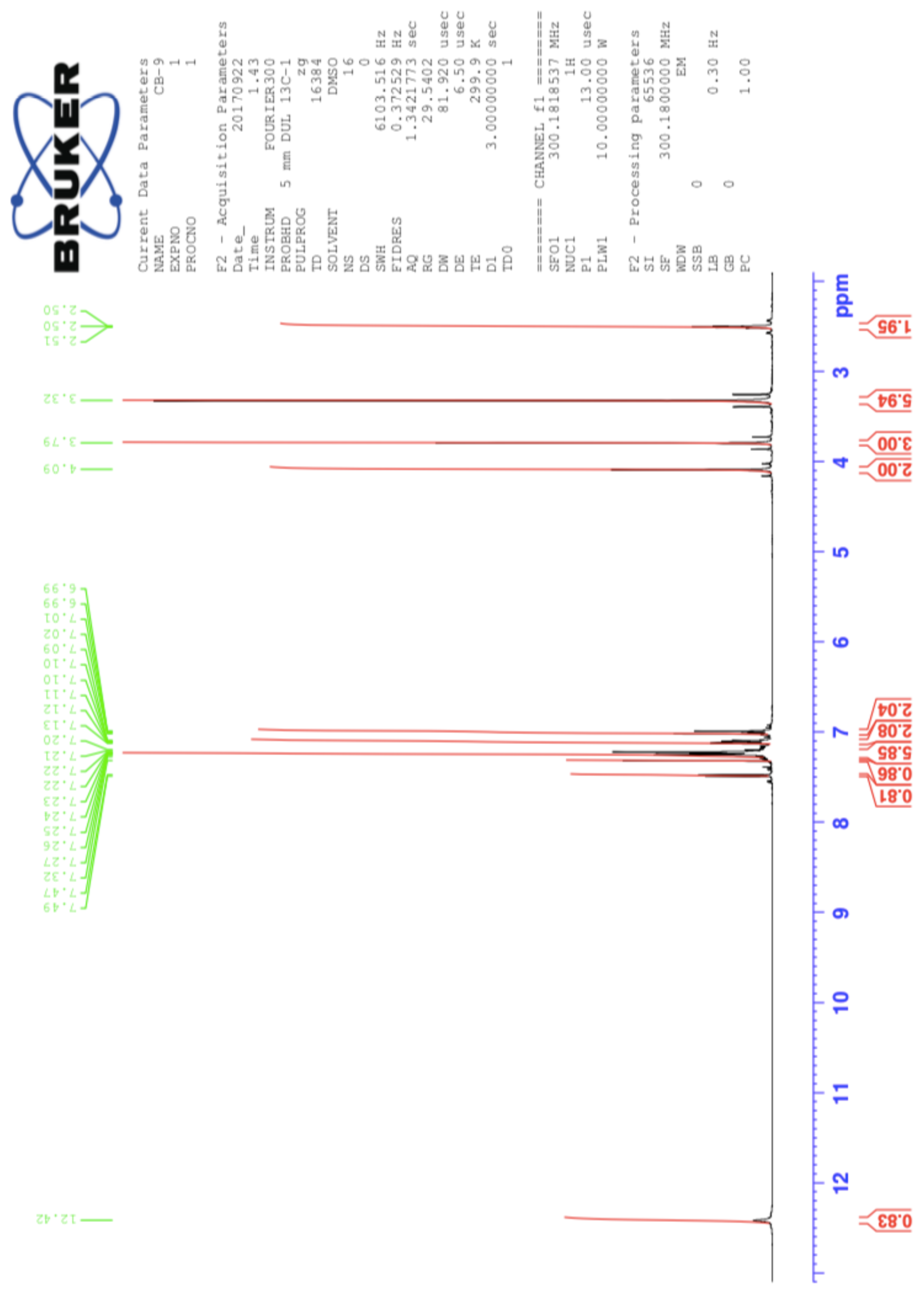

Supplement: Figure S.7 — 1H-NMR spectrum of Compound 7 [file turkjchem-45-6-1841s7.tif]

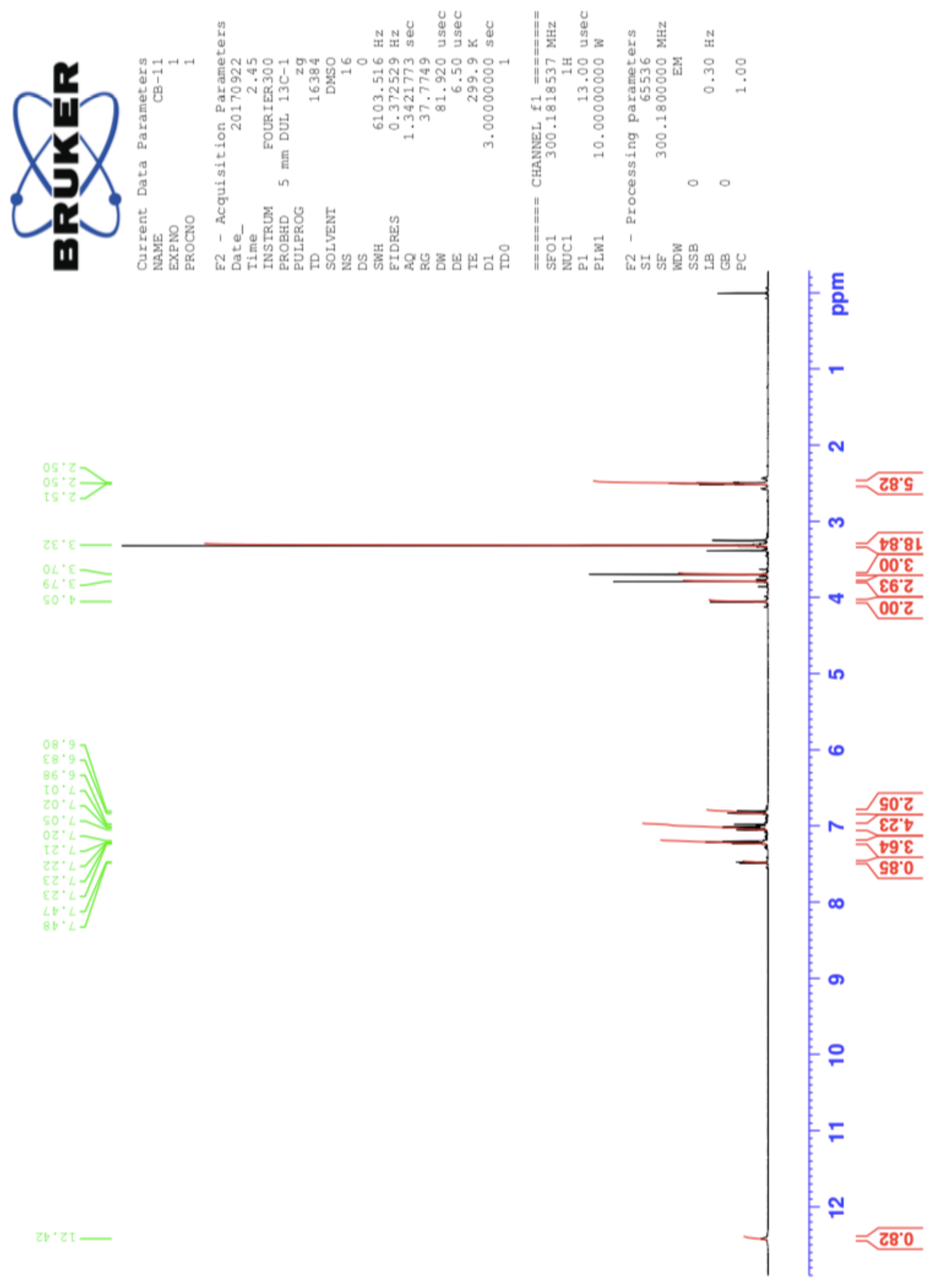

Supplement: Figure S.8 — 1H-NMR spectrum of Compound 8 [file turkjchem-45-6-1841s8.tif]

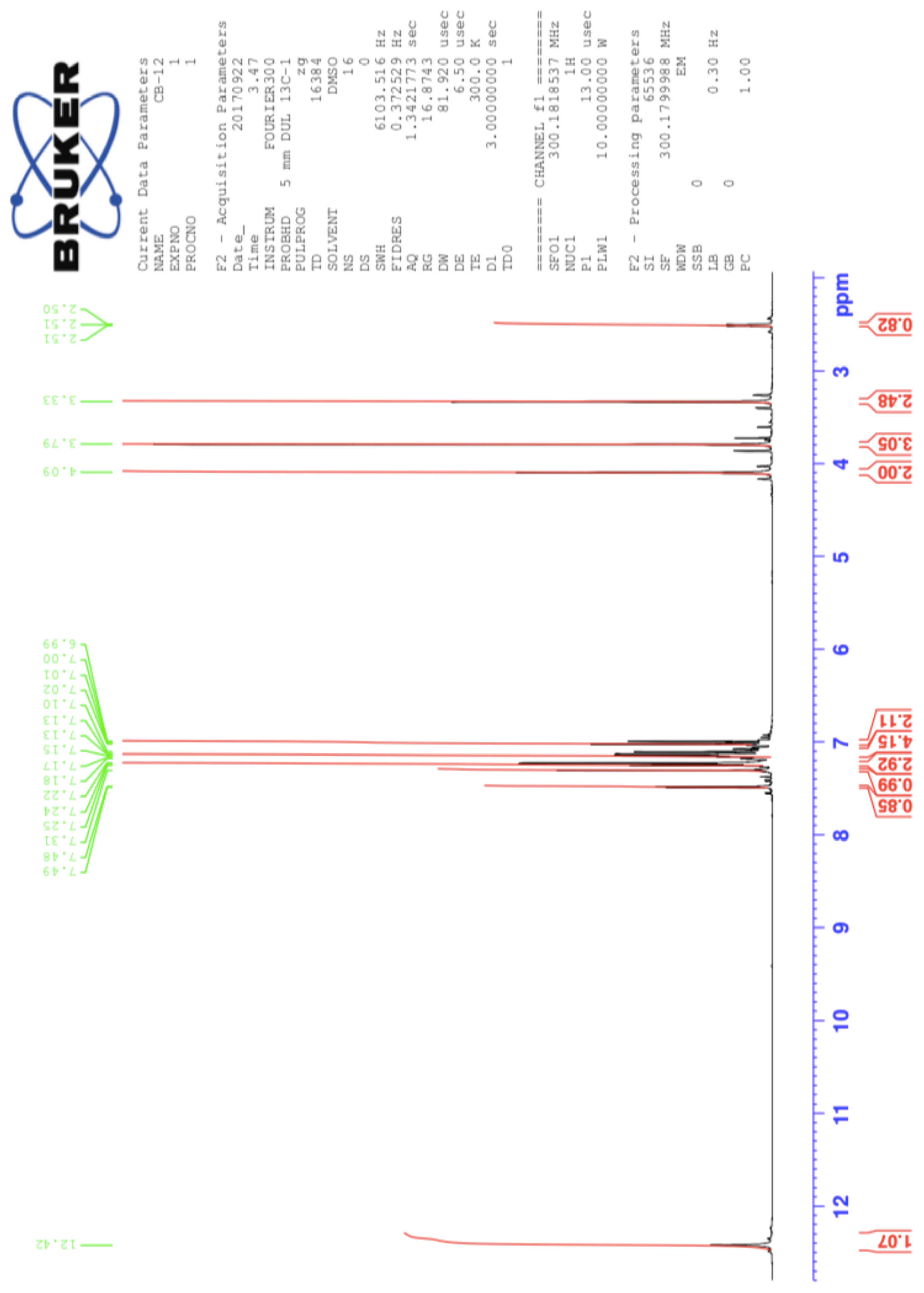

Supplement: Figure S.9 — 1H-NMR spectrum of Compound 9 [file turkjchem-45-6-1841s9.tif]

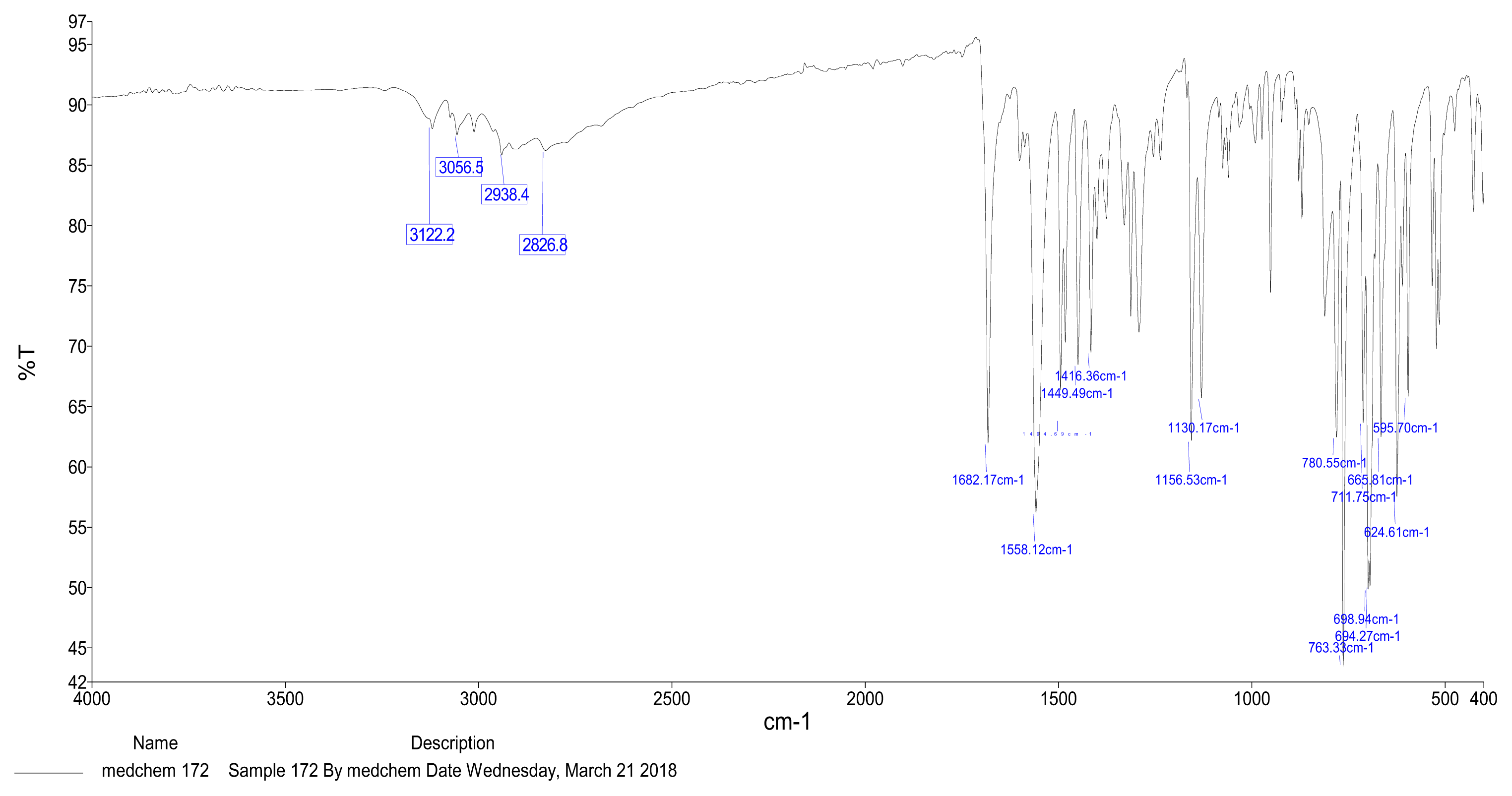

Supplement: Figure S.10 — IR spectrum of Compound 1 [file turkjchem-45-6-1841s10.tif]

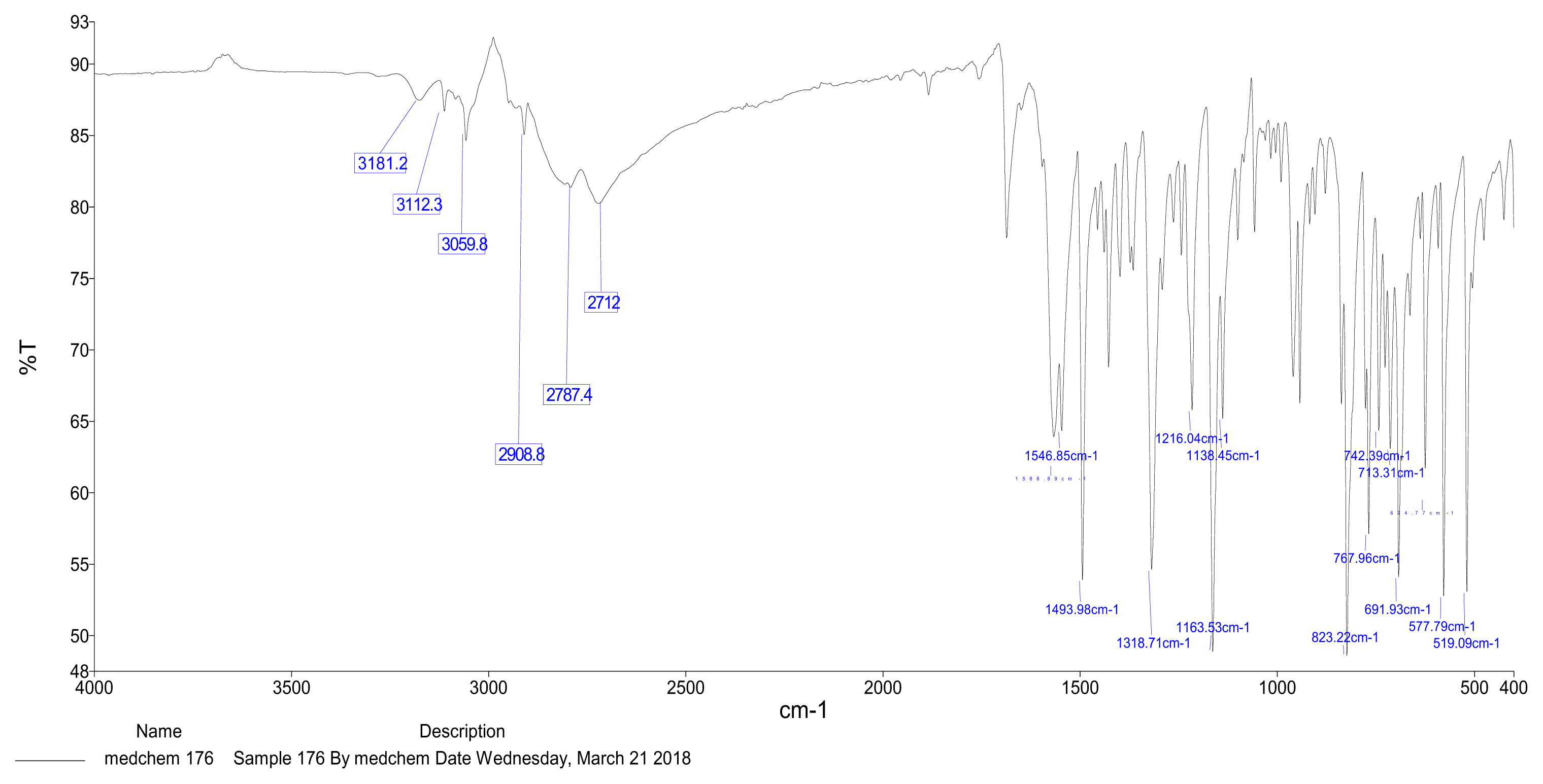

Supplement: Figure S.11 — IR spectrum of Compound 2 [file turkjchem-45-6-1841s11.tif]

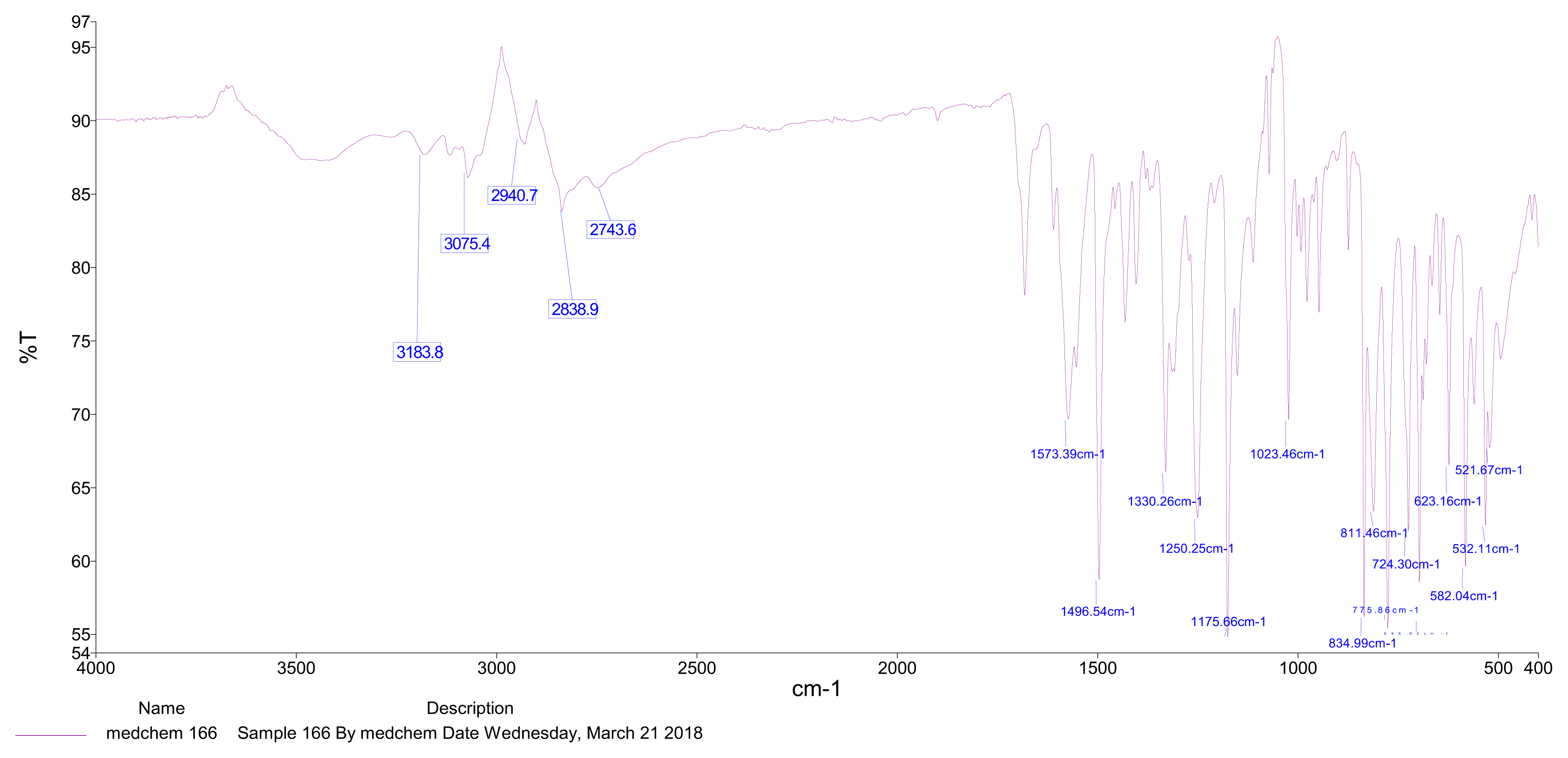

Supplement: Figure S.12 — IR spectrum of Compound 3 [file turkjchem-45-6-1841s12.tif]

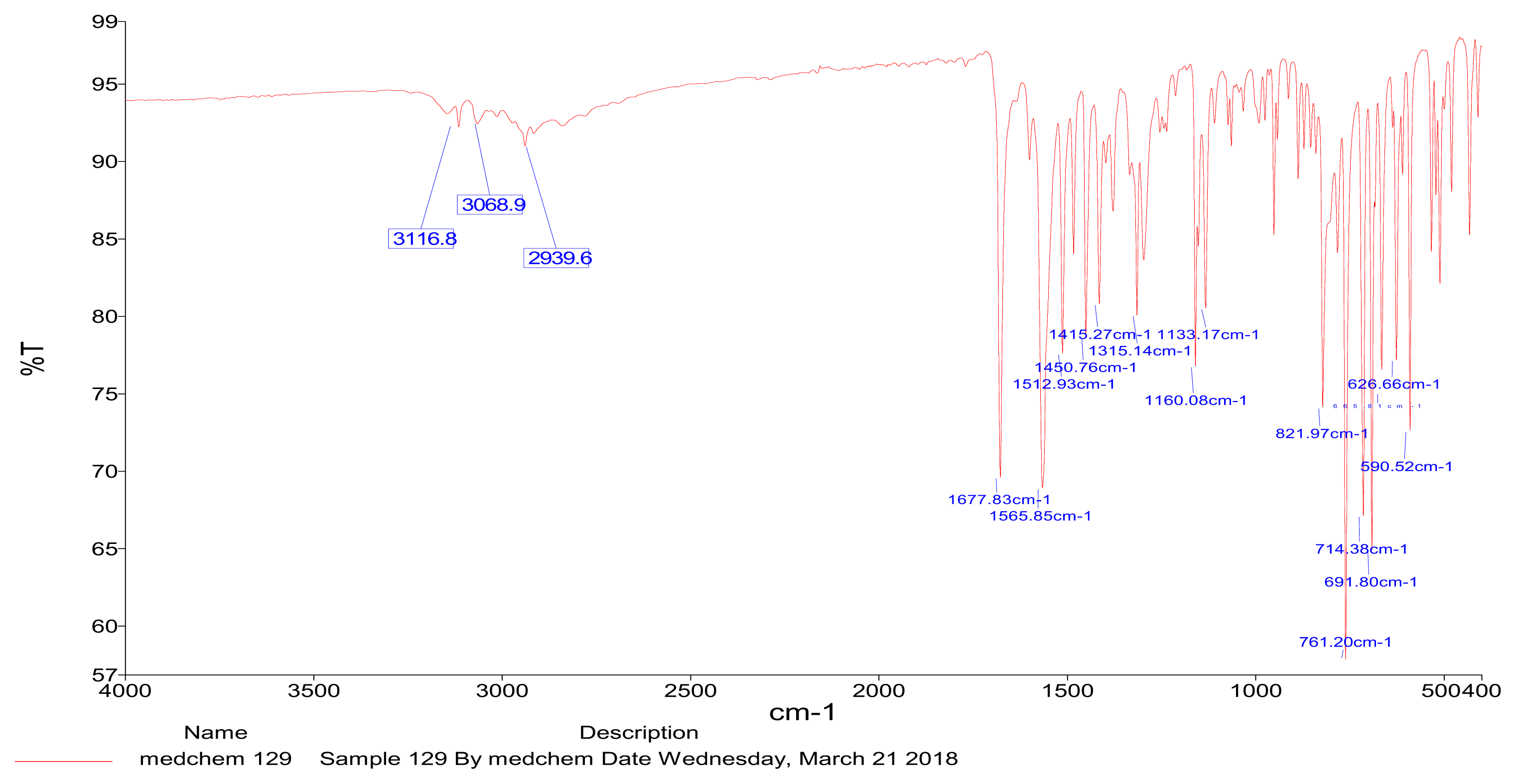

Supplement: Figure S.13 — IR spectrum of Compound 4 [file turkjchem-45-6-1841s13.tif]

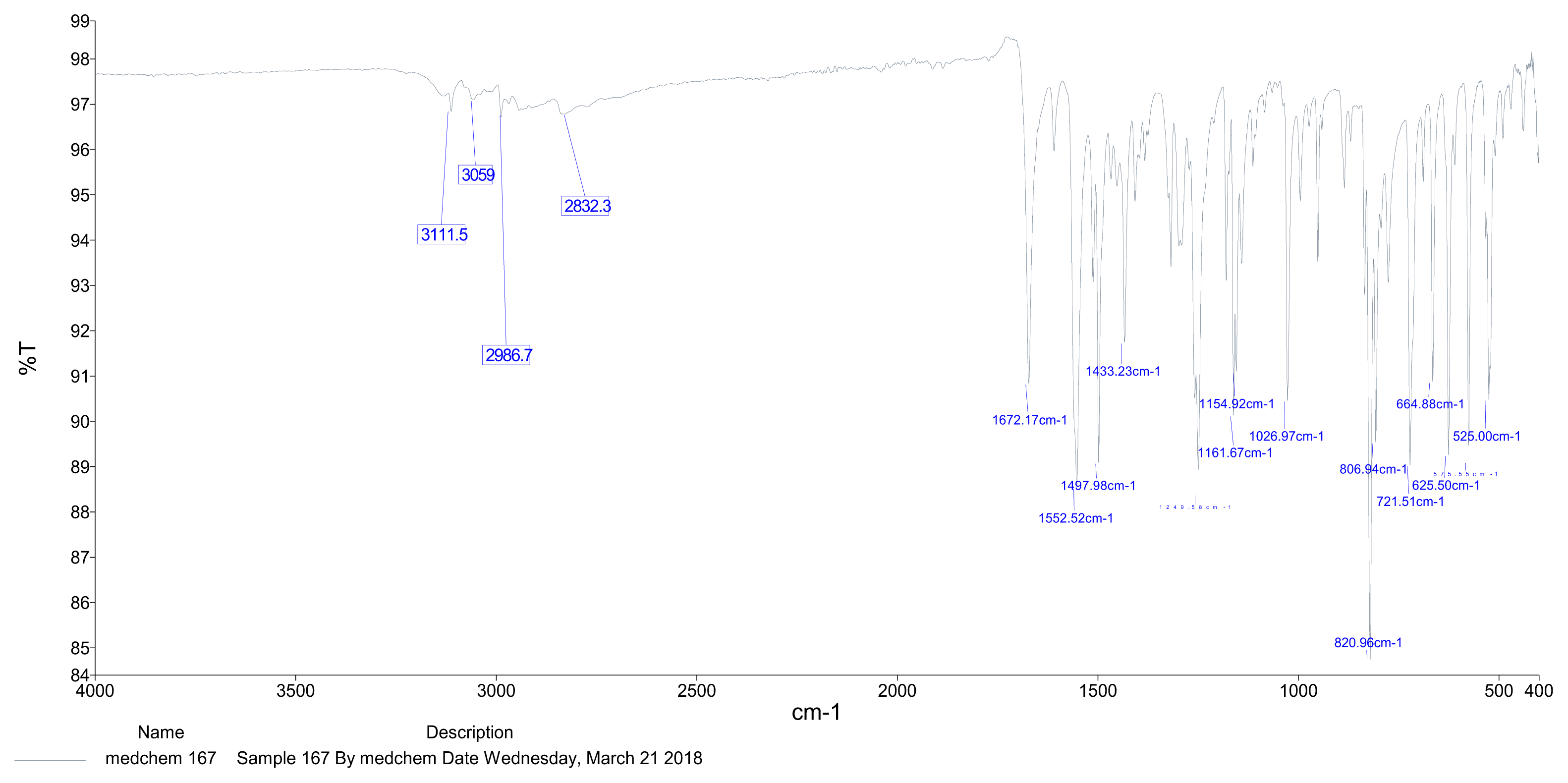

Supplement: Figure S.14 — IR spectrum of Compound 5 [file turkjchem-45-6-1841s14.tif]

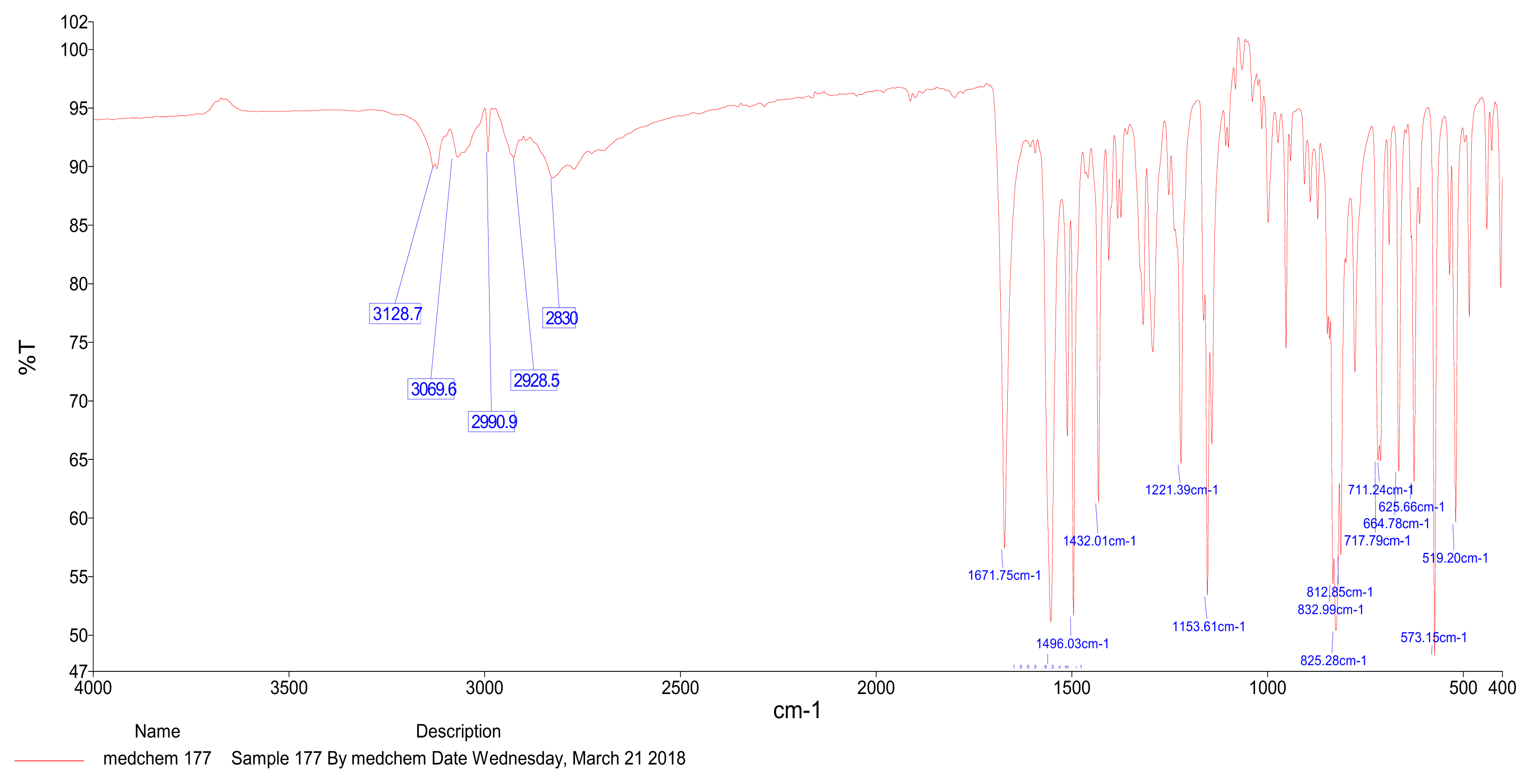

Supplement: Figure S.15 — IR spectrum of Compound 6 [file turkjchem-45-6-1841s15.tif]

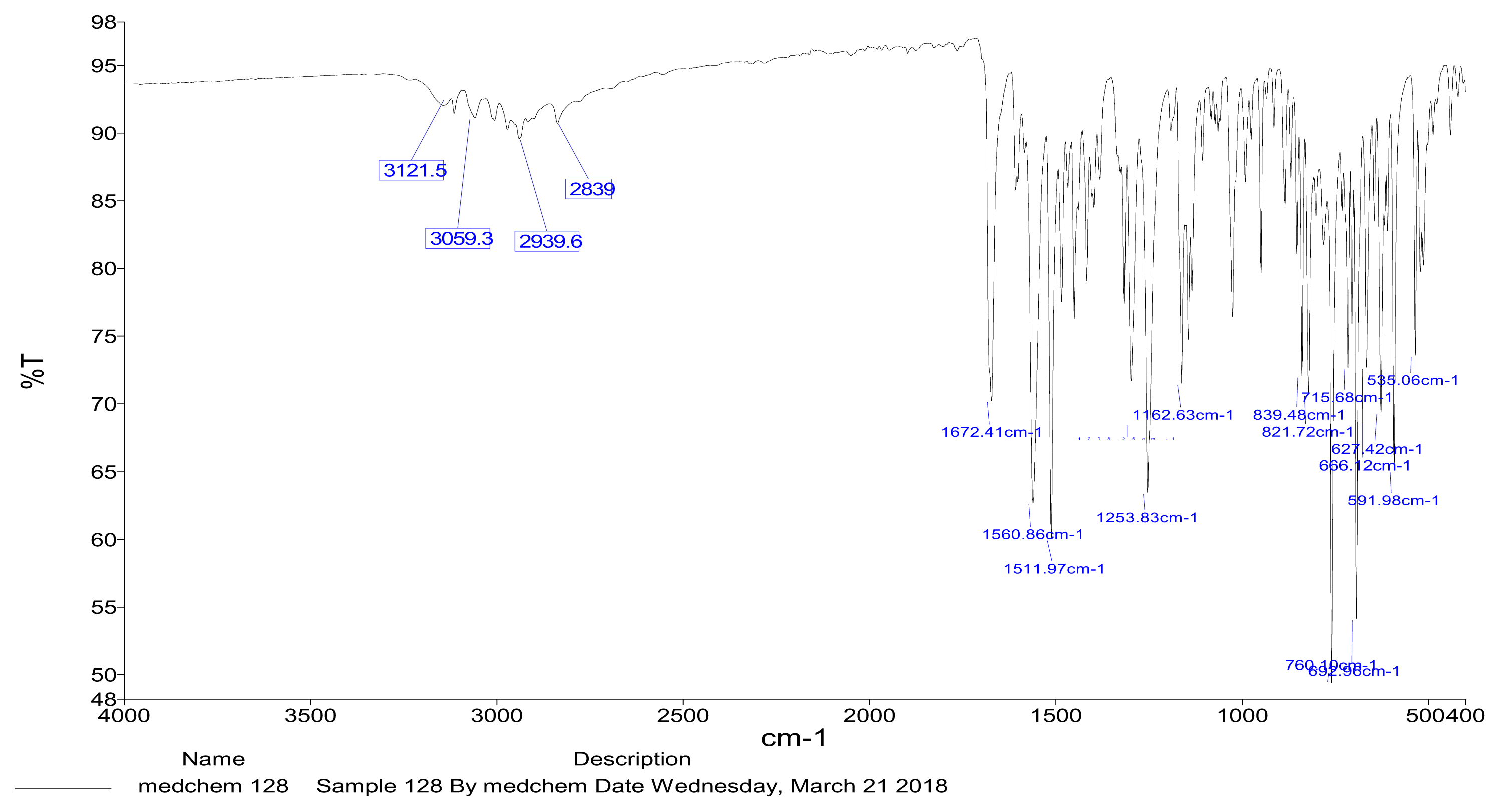

Supplement: Figure S.16 — IR spectrum of Compound 7 [file turkjchem-45-6-1841s16.tif]

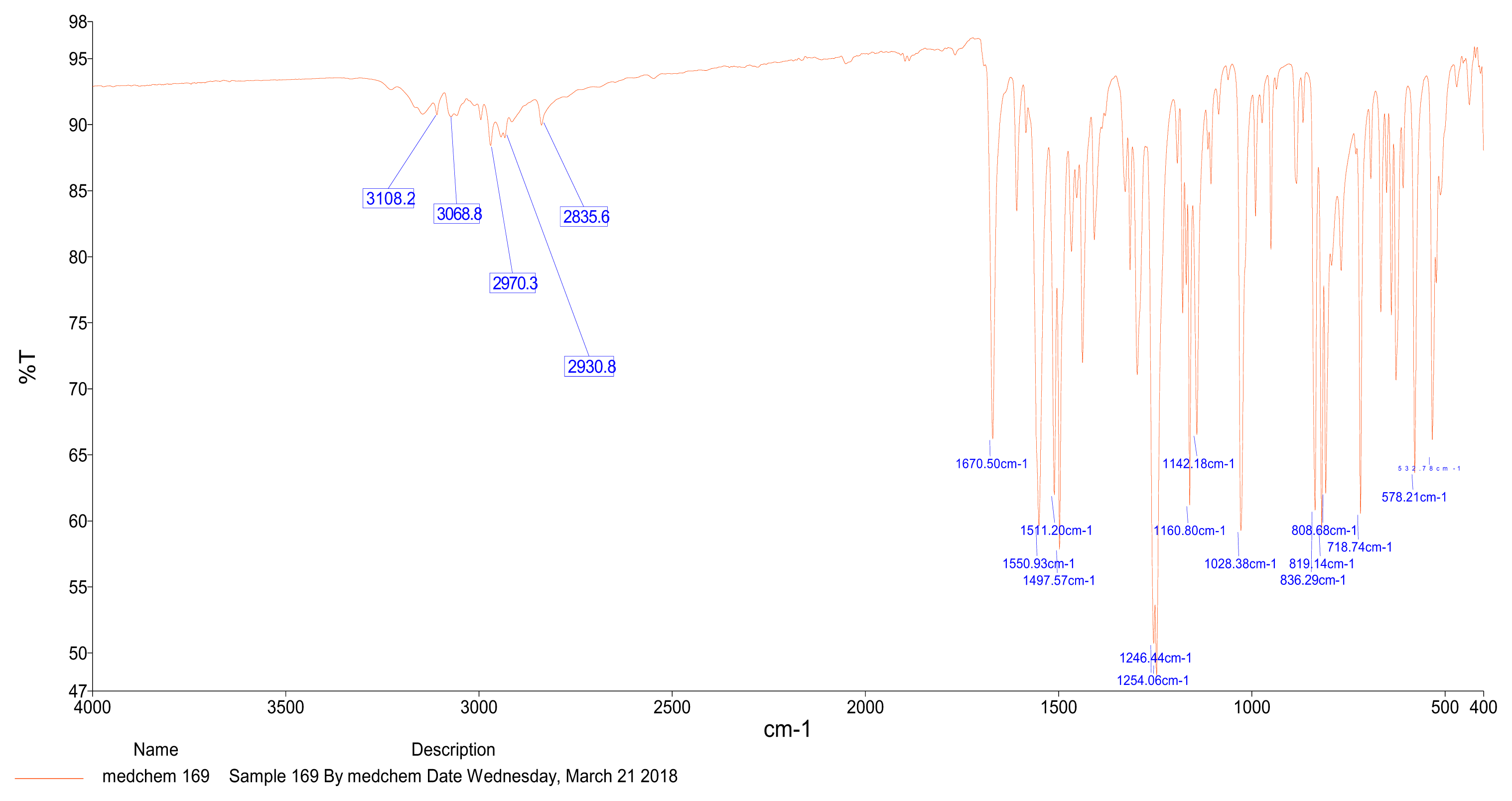

Supplement: Figure S.17 — IR spectrum of Compound 8 [file turkjchem-45-6-1841s17.tif]

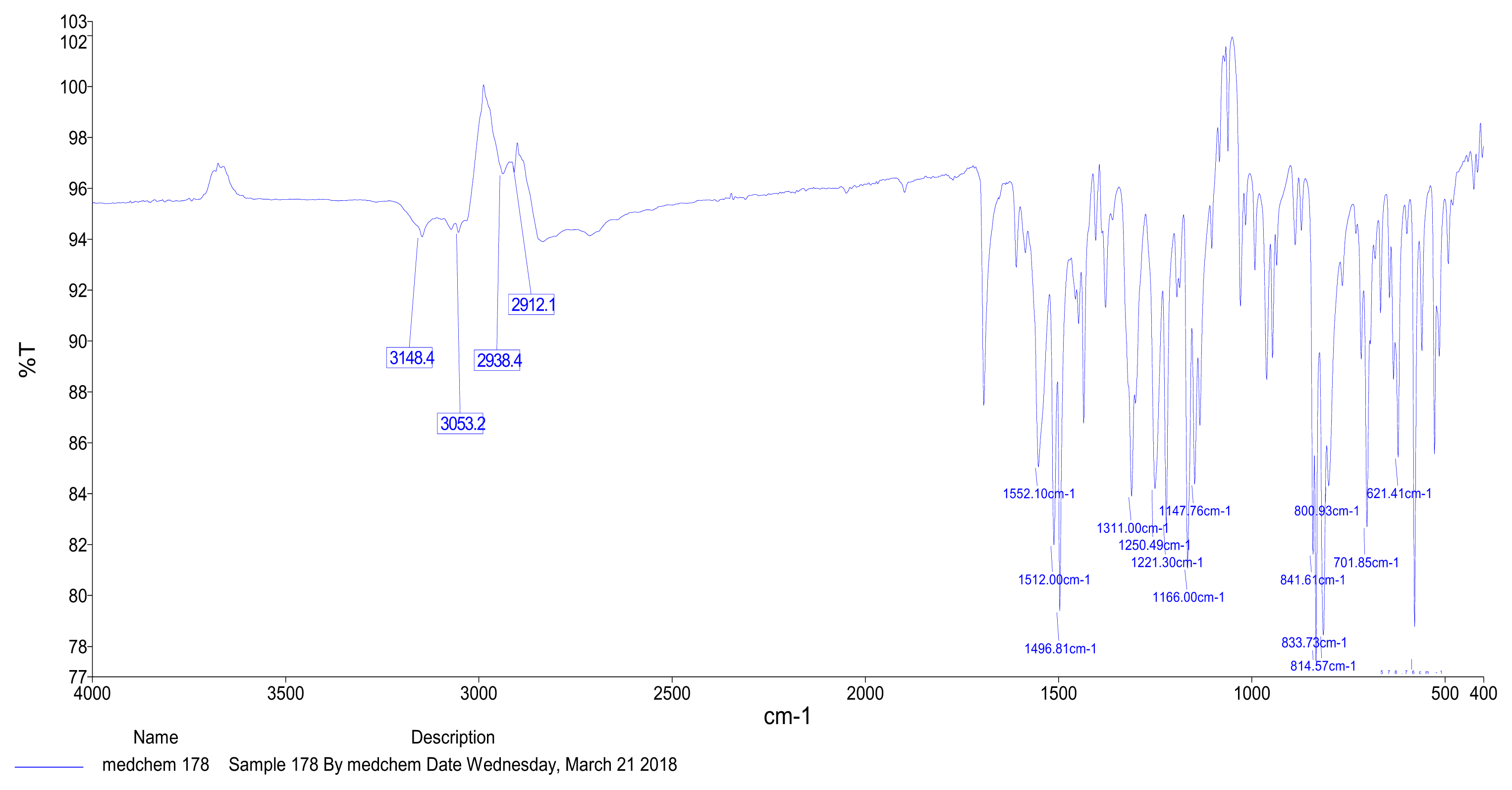

Supplement: Figure S.18 — IR spectrum of Compound 9 [file turkjchem-45-6-1841s18.tif]

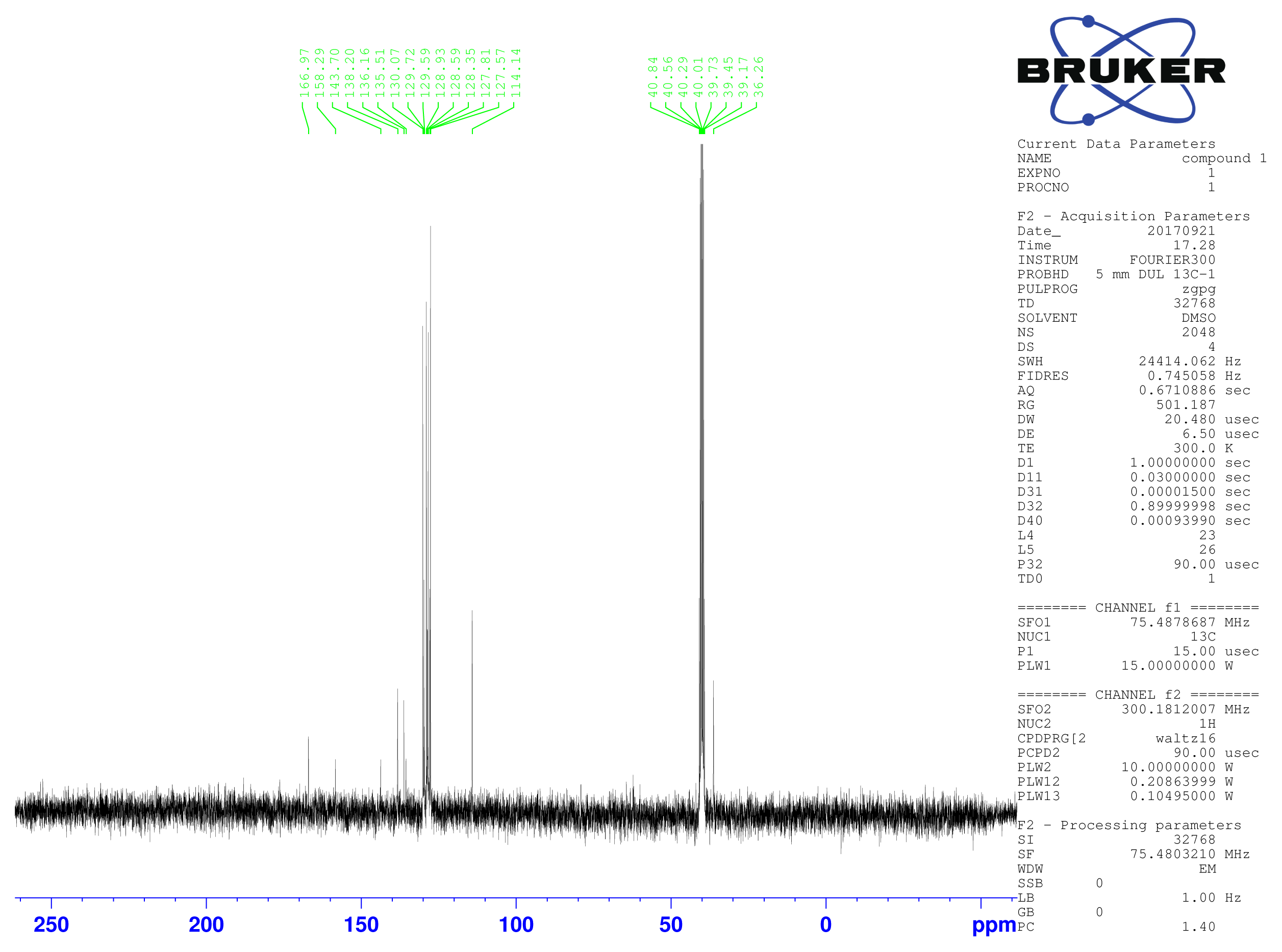

Supplement: Figure S.19 — 13C NMR spectrum of Compound 1 [file turkjchem-45-6-1841s19.tif]

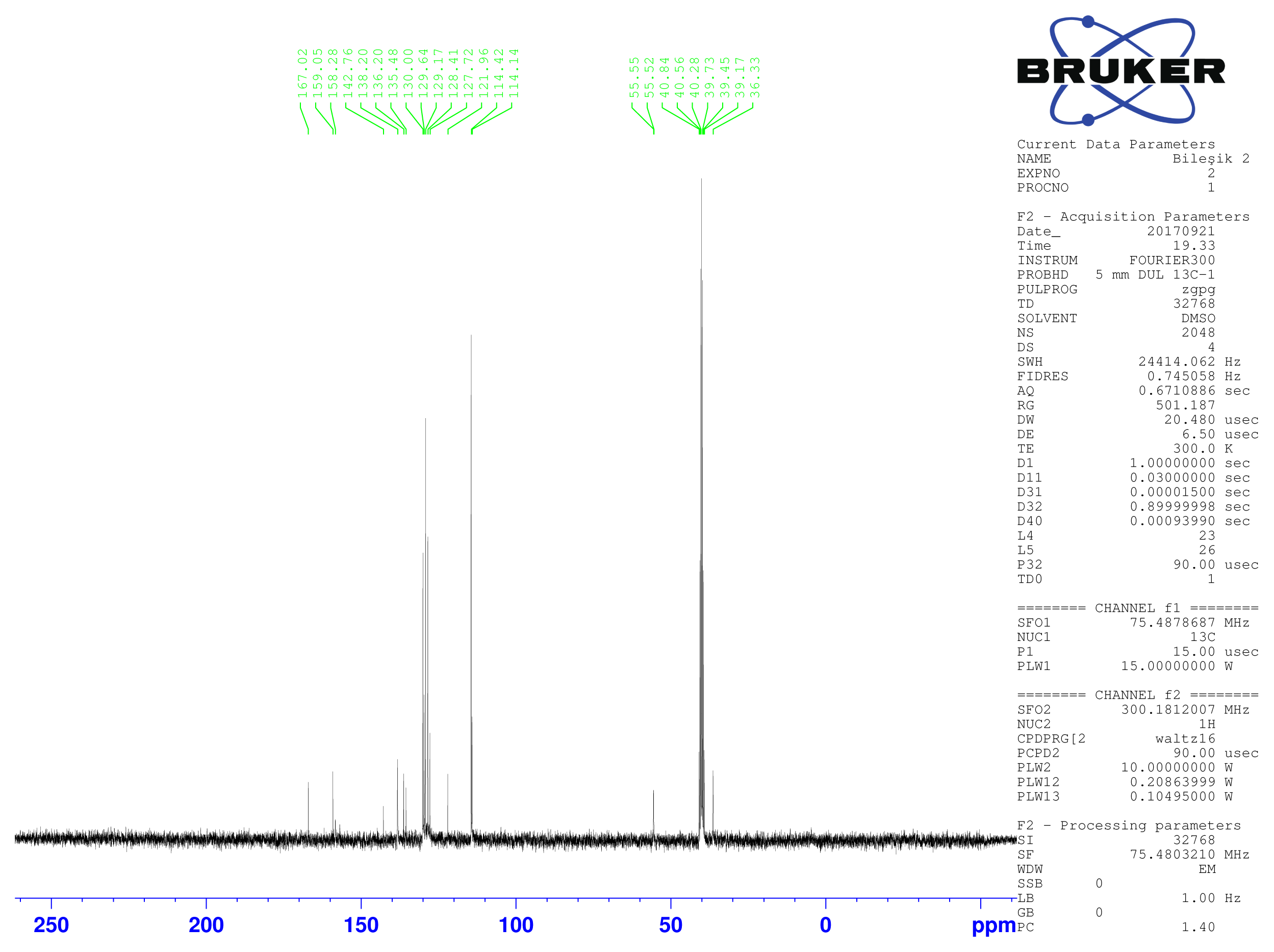

Supplement: Figure S.20 — 13C NMR spectrum of Compound 2 [file turkjchem-45-6-1841s20.tif]

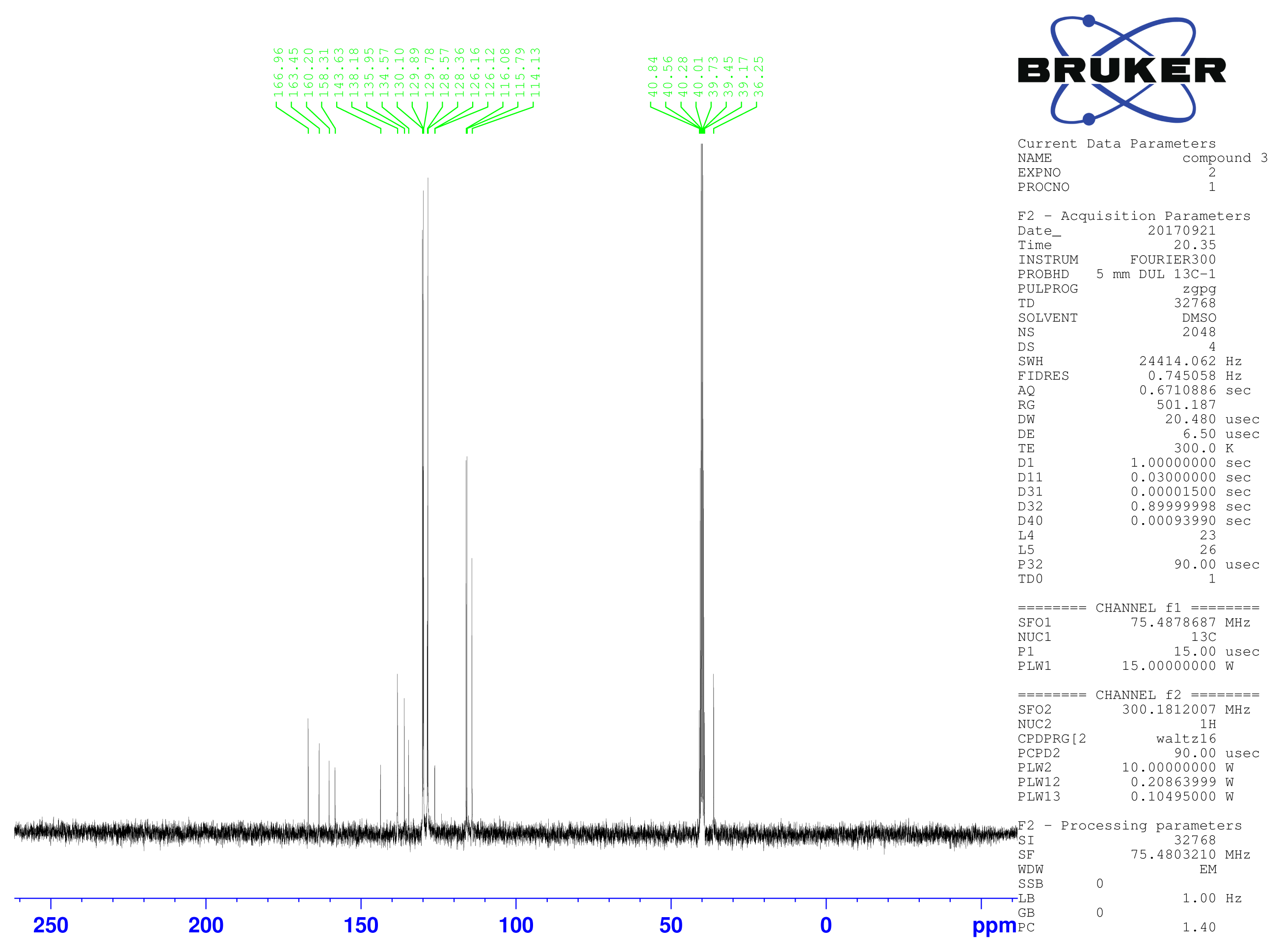

Supplement: Figure S.21 — 13C NMR spectrum of Compound 3 [file turkjchem-45-6-1841s21.tif]

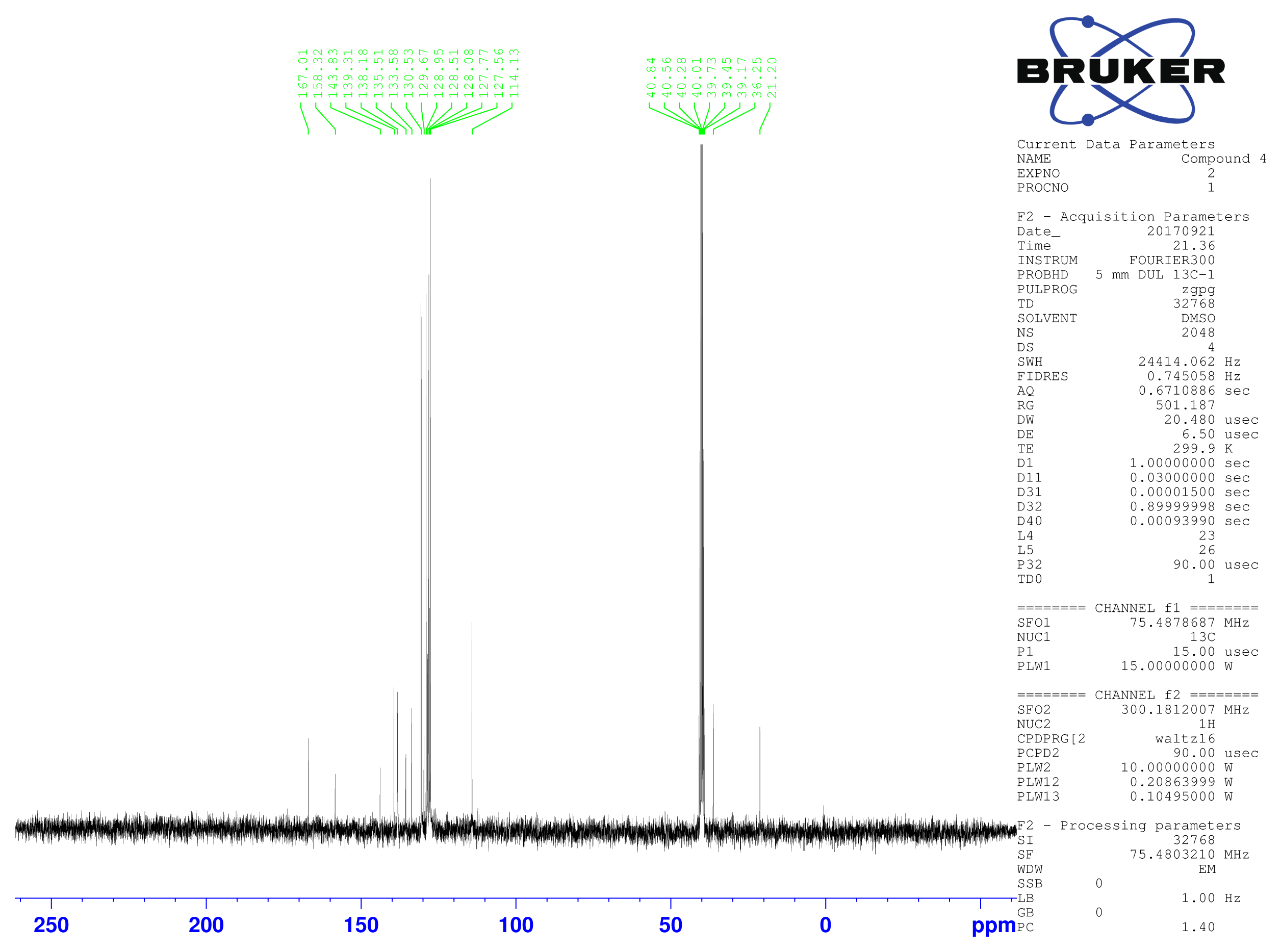

Supplement: Figure S.22 — 13C NMR spectrum of Compound 4 [file turkjchem-45-6-1841s22.tif]

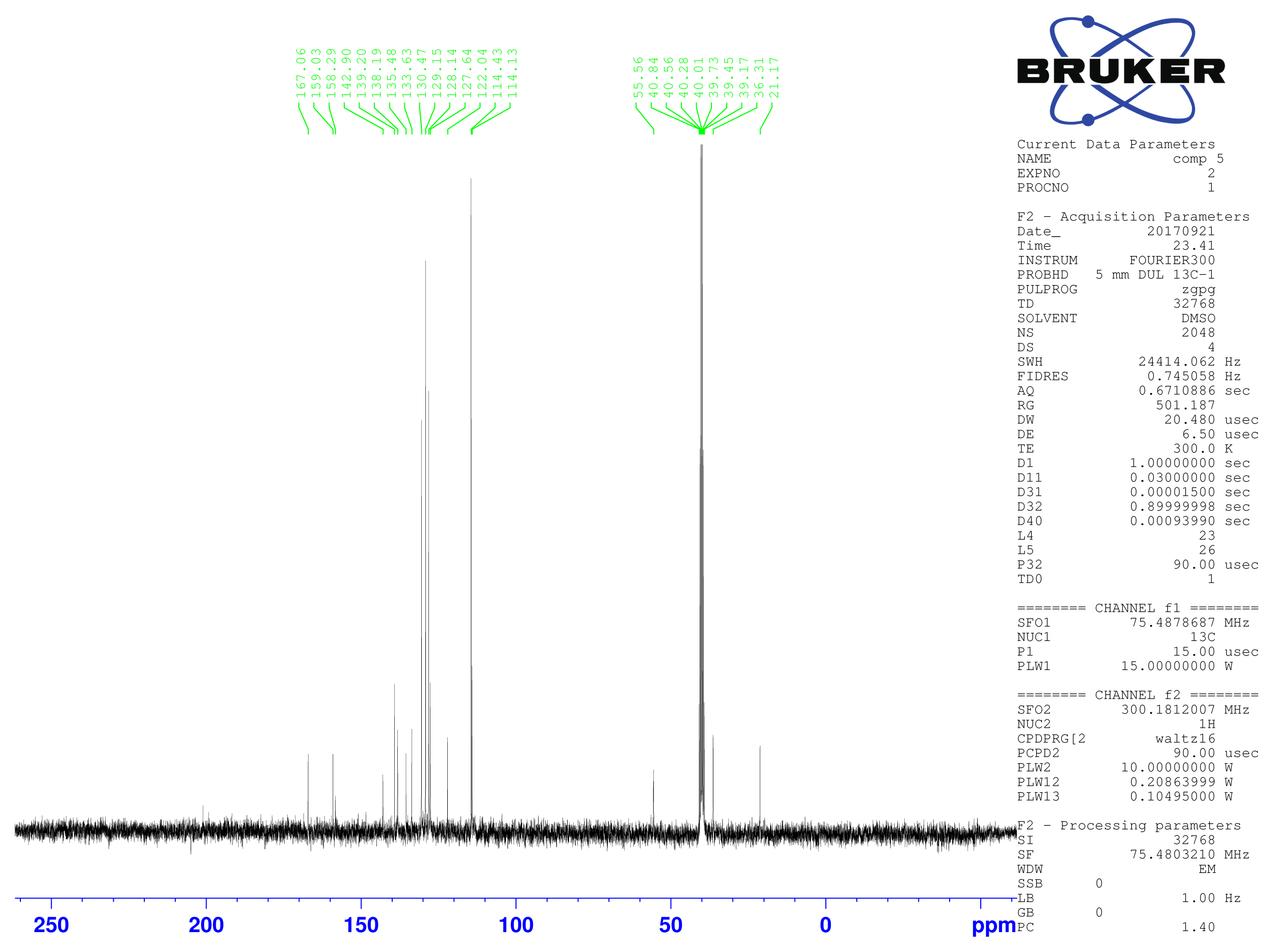

Supplement: Figure S.23 — 13C NMR spectrum of Compound 5 [file turkjchem-45-6-1841s23.tif]

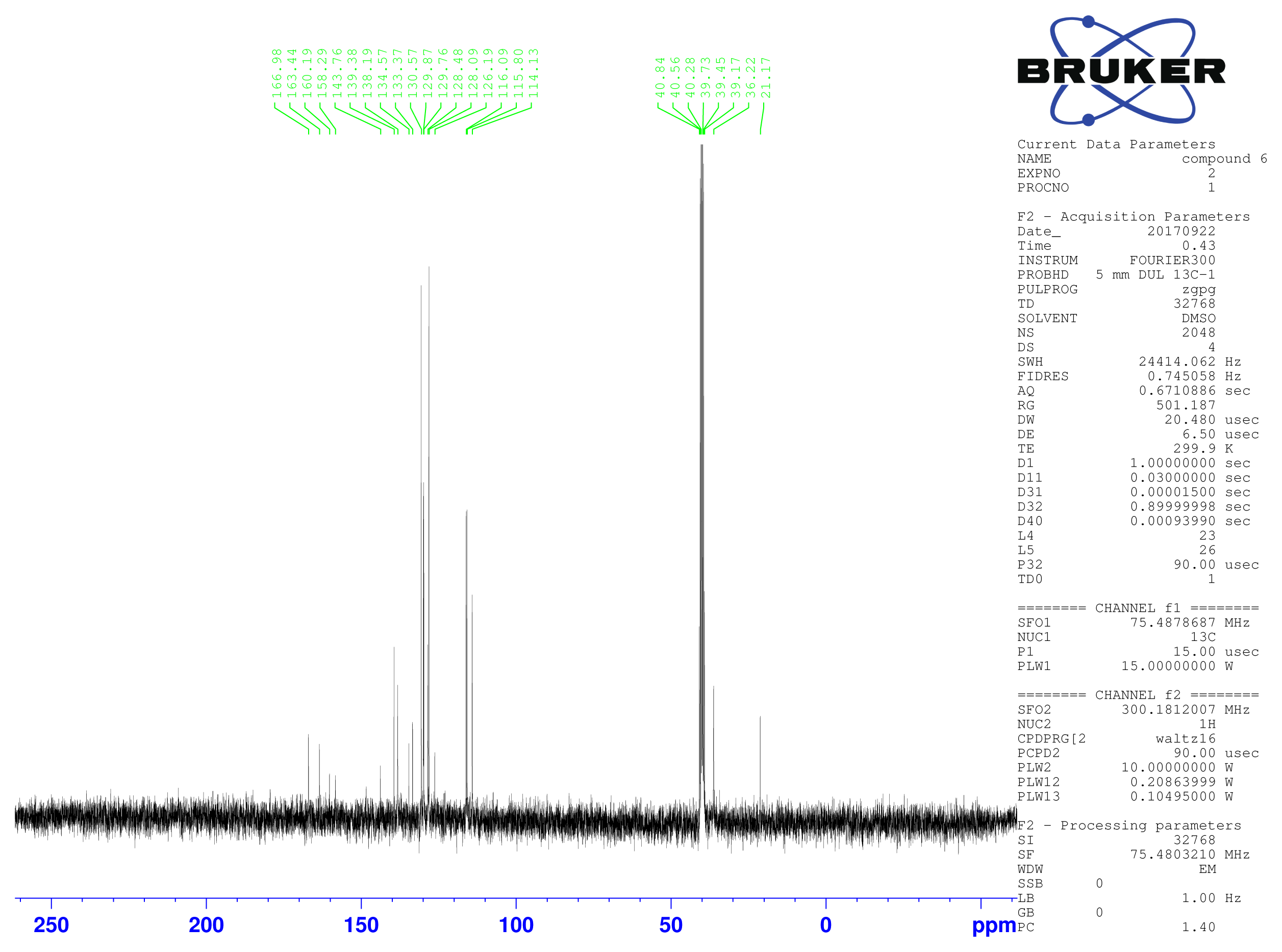

Supplement: Figure S.24 — 13C NMR spectrum of Compound 6 [file turkjchem-45-6-1841s24.tif]

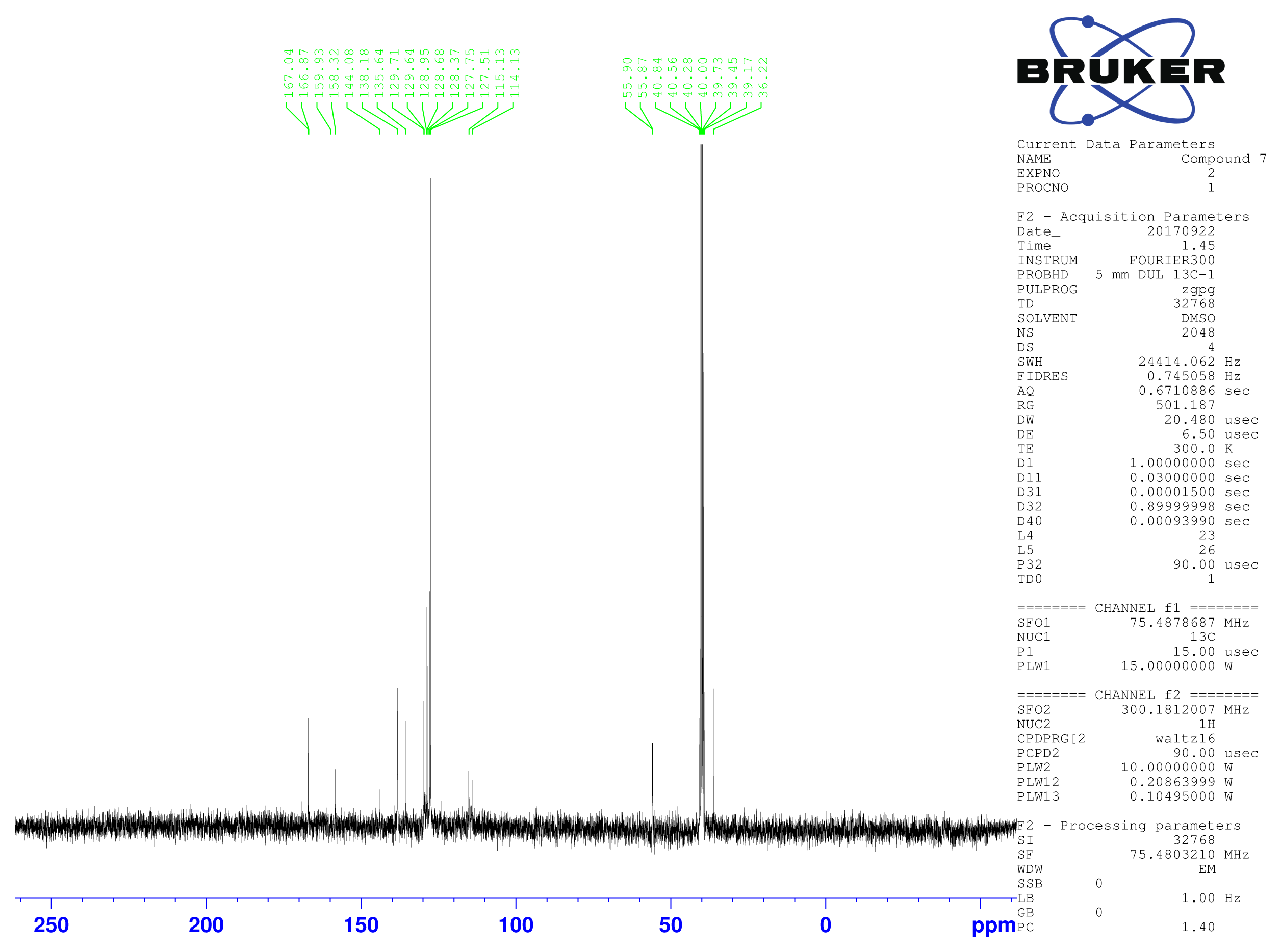

Supplement: Figure S.25 — 13C NMR spectrum of Compound 7 [file turkjchem-45-6-1841s25.tif]

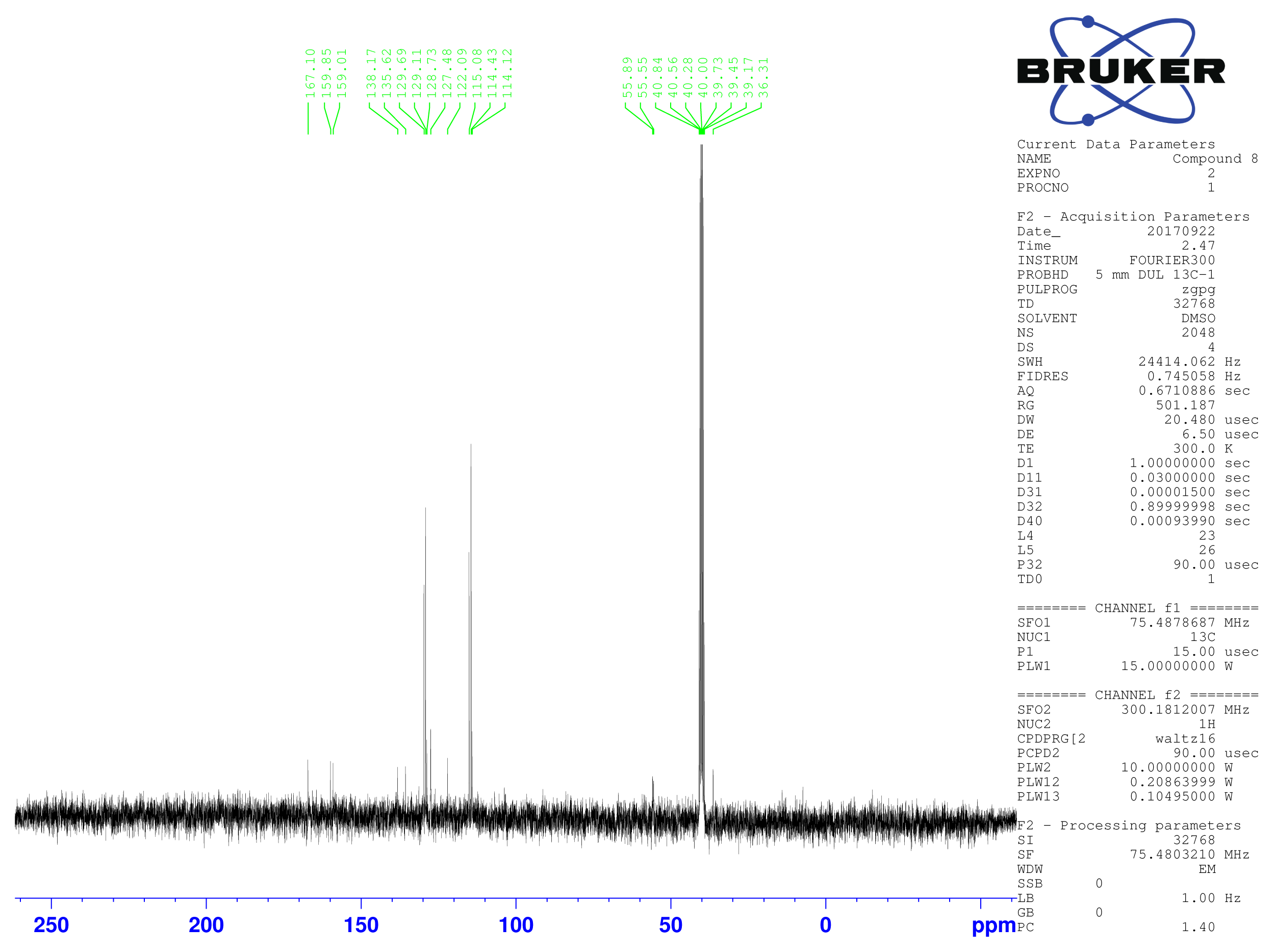

Supplement: Figure S.26 — 13C NMR spectrum of Compound 8 [file turkjchem-45-6-1841s26.tif]

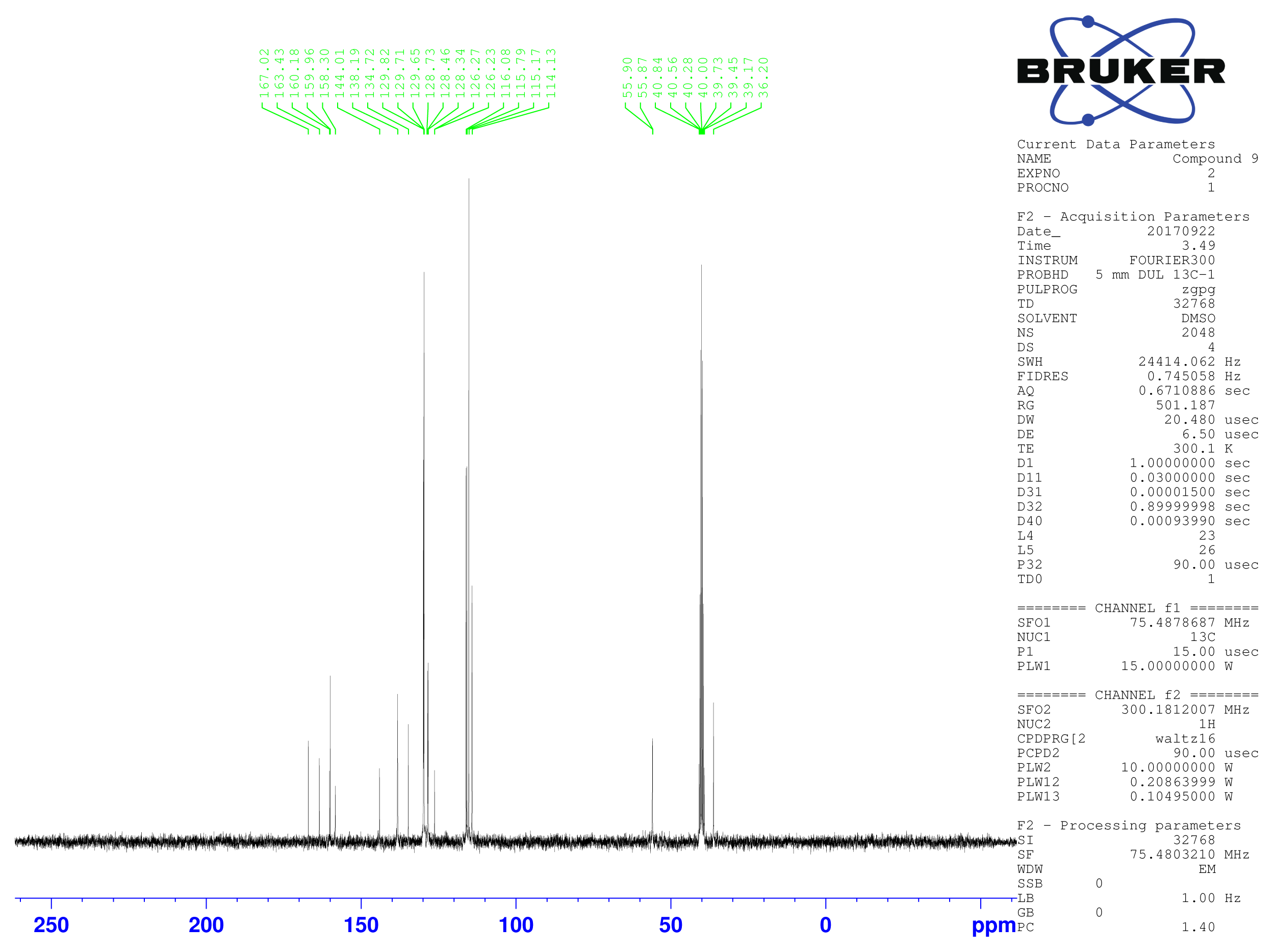

Supplement: Figure S.27 — 13C NMR spectrum of Compound 9 [file turkjchem-45-6-1841s27.tif]

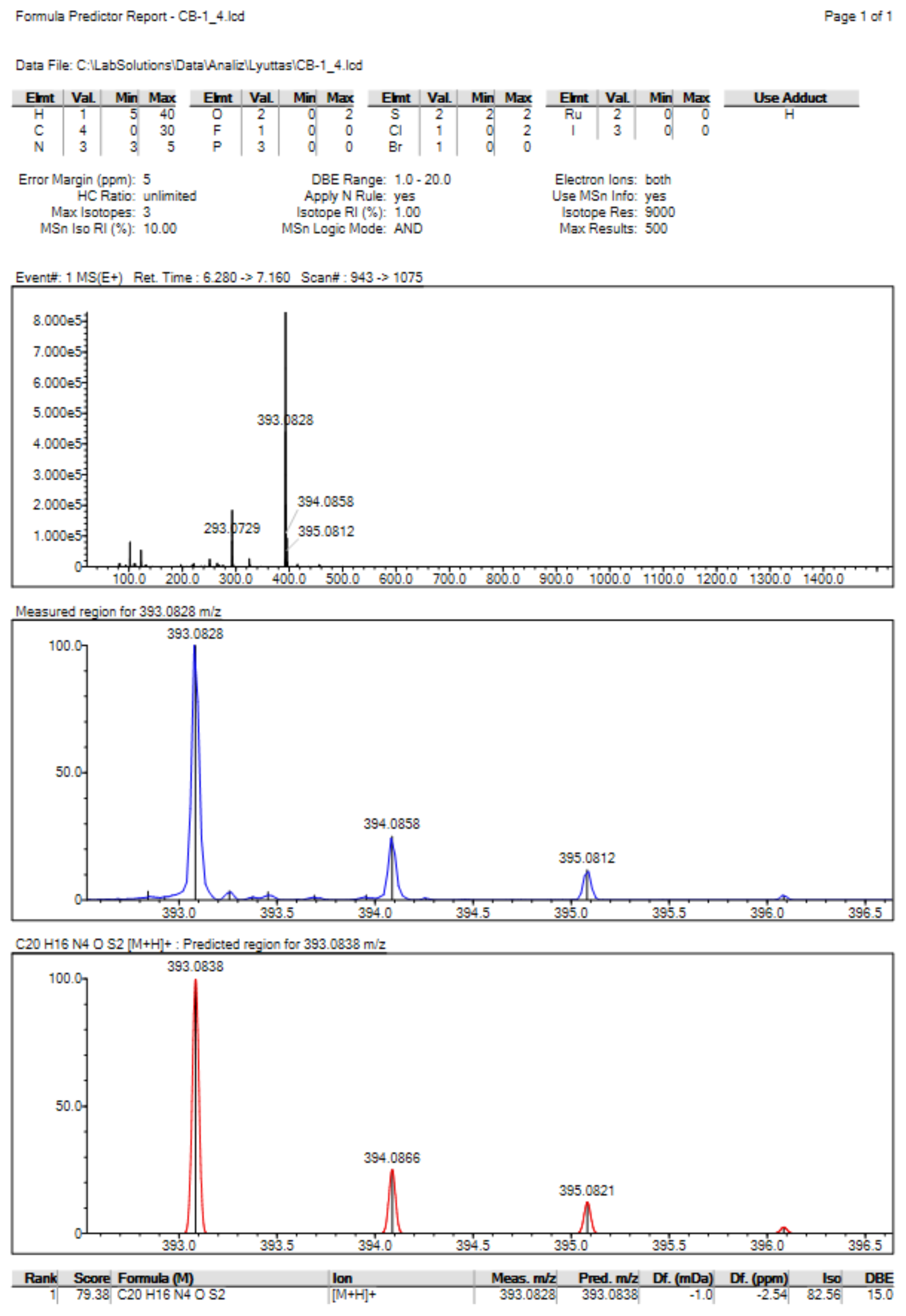

Supplement: Figure S.28 — HRMS spectrum of Compound 1 [file turkjchem-45-6-1841s28.tif]

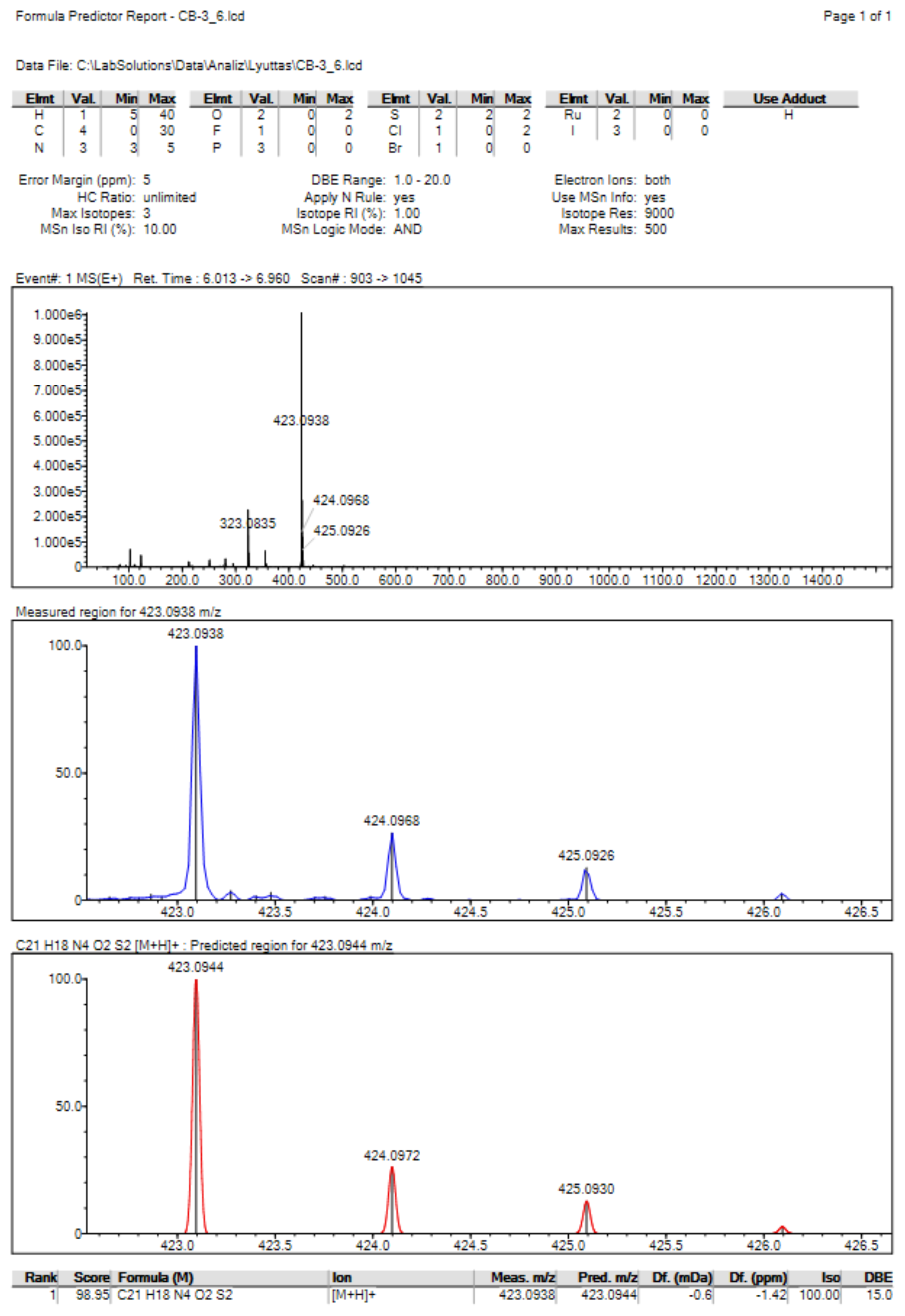

Supplement: Figure S.29 — HRMS spectrum of Compound 2 [file turkjchem-45-6-1841s29.tif]

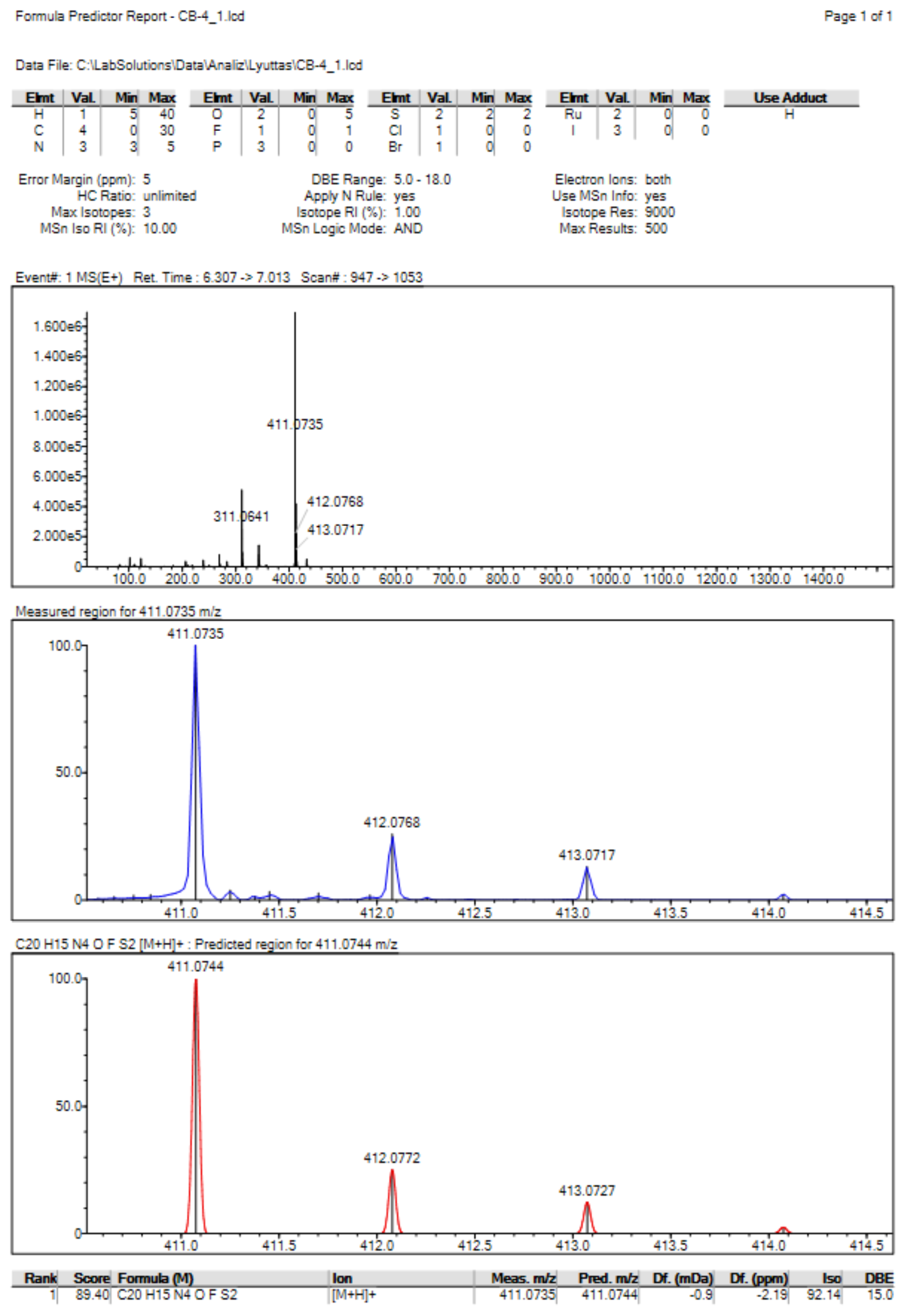

Supplement: Figure S.30 — HRMS spectrum of Compound 3 [file turkjchem-45-6-1841s30.tif]

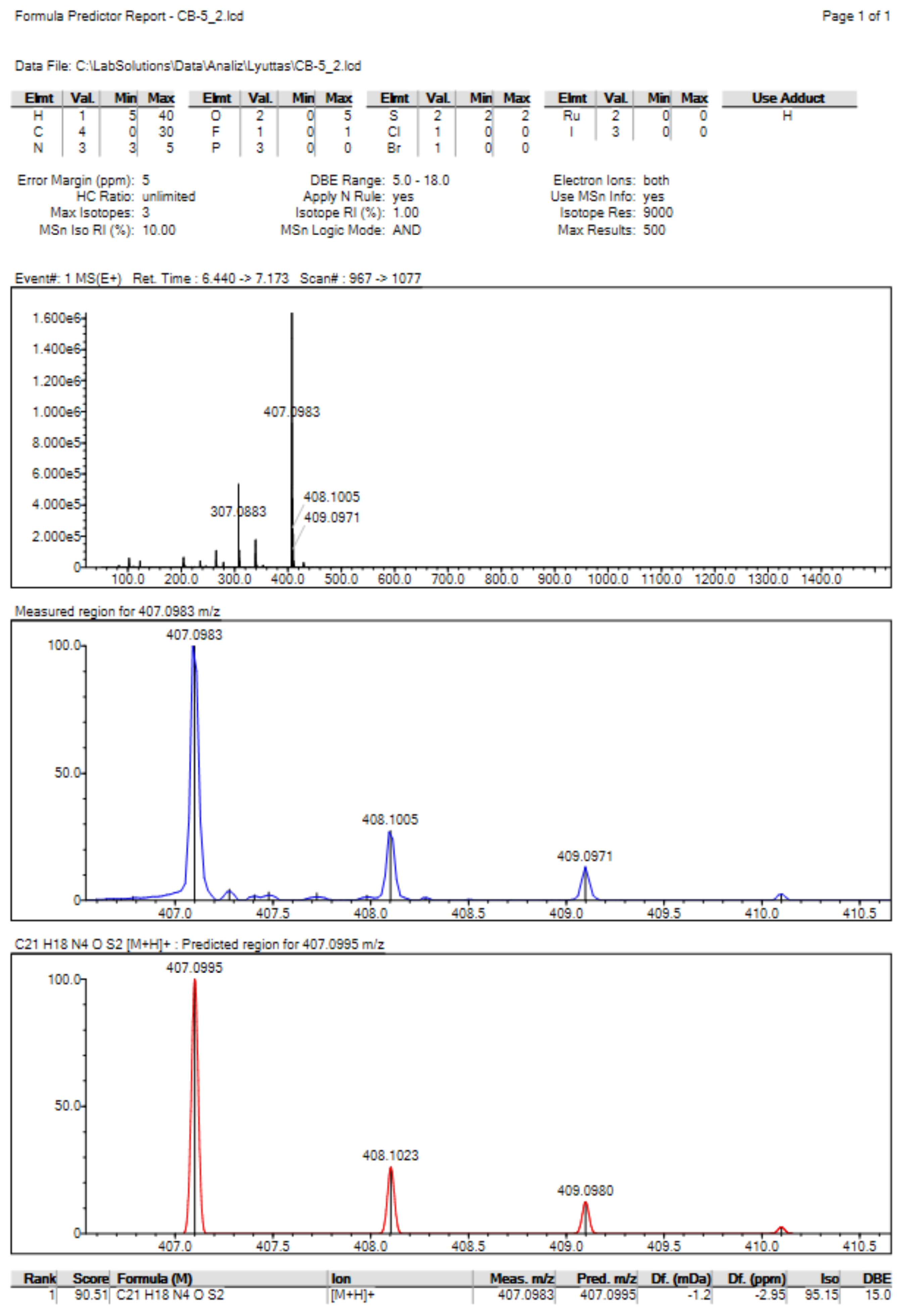

Supplement: Figure S.31 — HRMS spectrum of Compound 4 [file turkjchem-45-6-1841s31.tif]

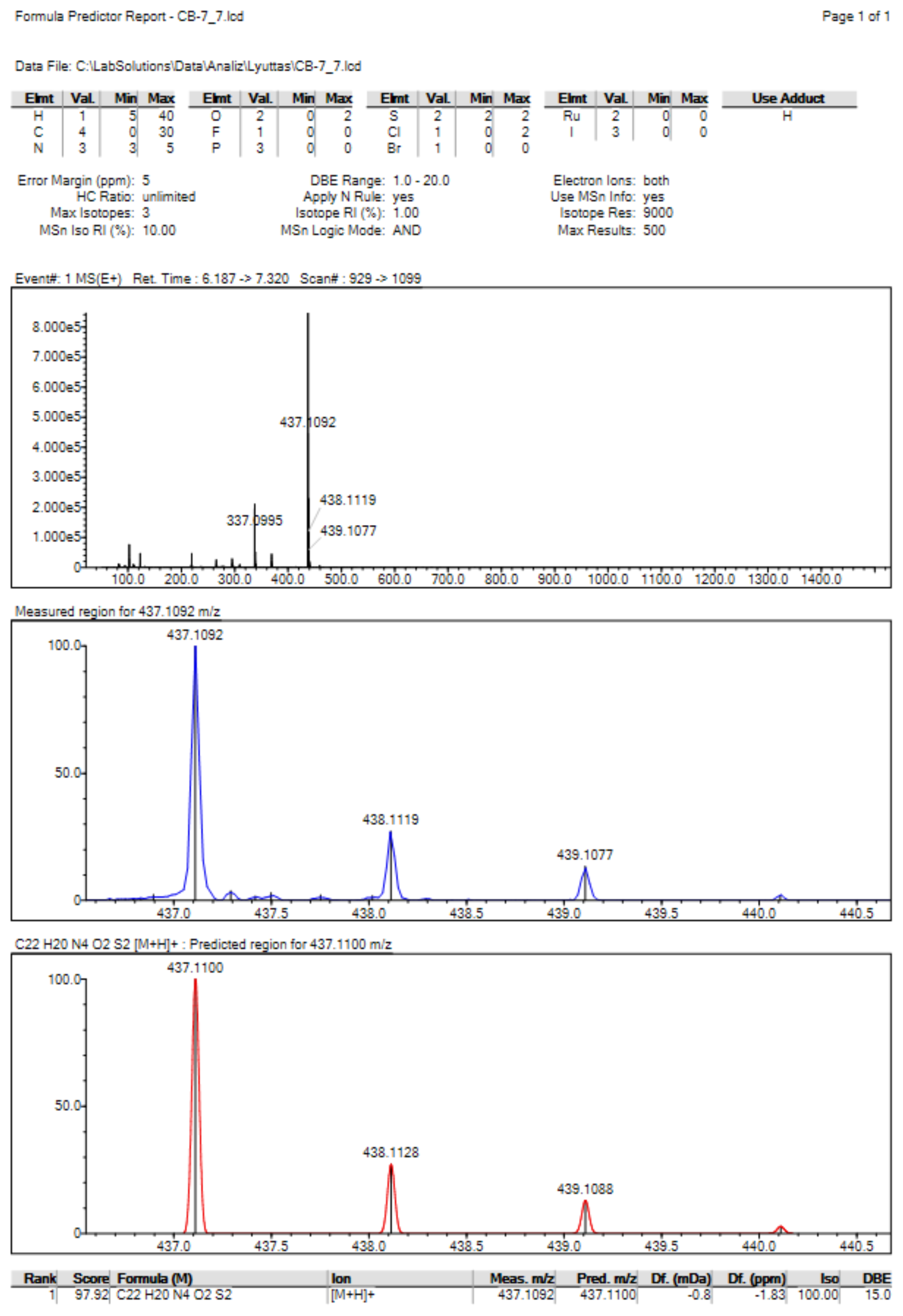

Supplement: Figure S.32 — HRMS spectrum of Compound 5 [file turkjchem-45-6-1841s32.tif]

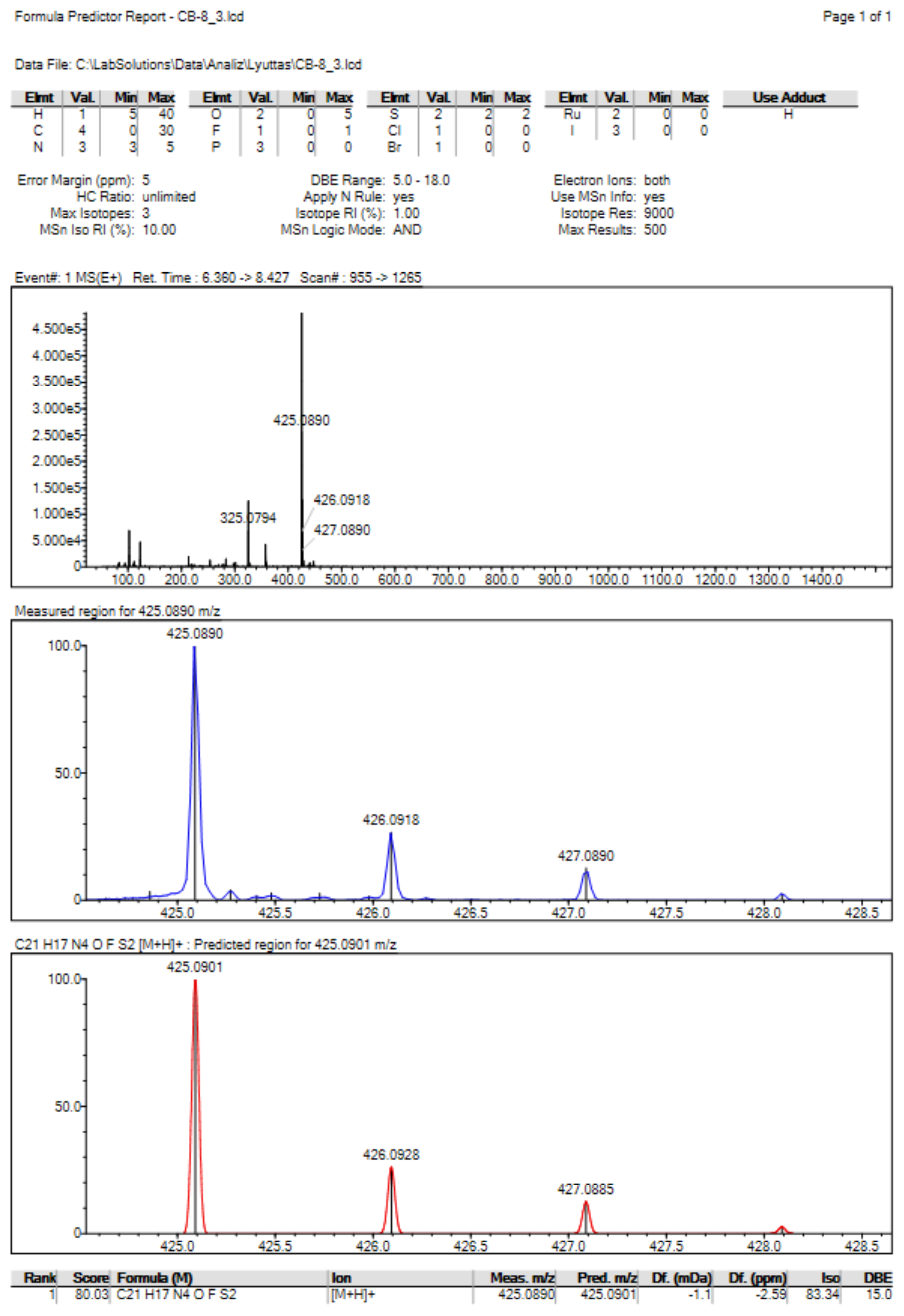

Supplement: Figure S.33 — HRMS spectrum of Compound 6 [file turkjchem-45-6-1841s33.tif]

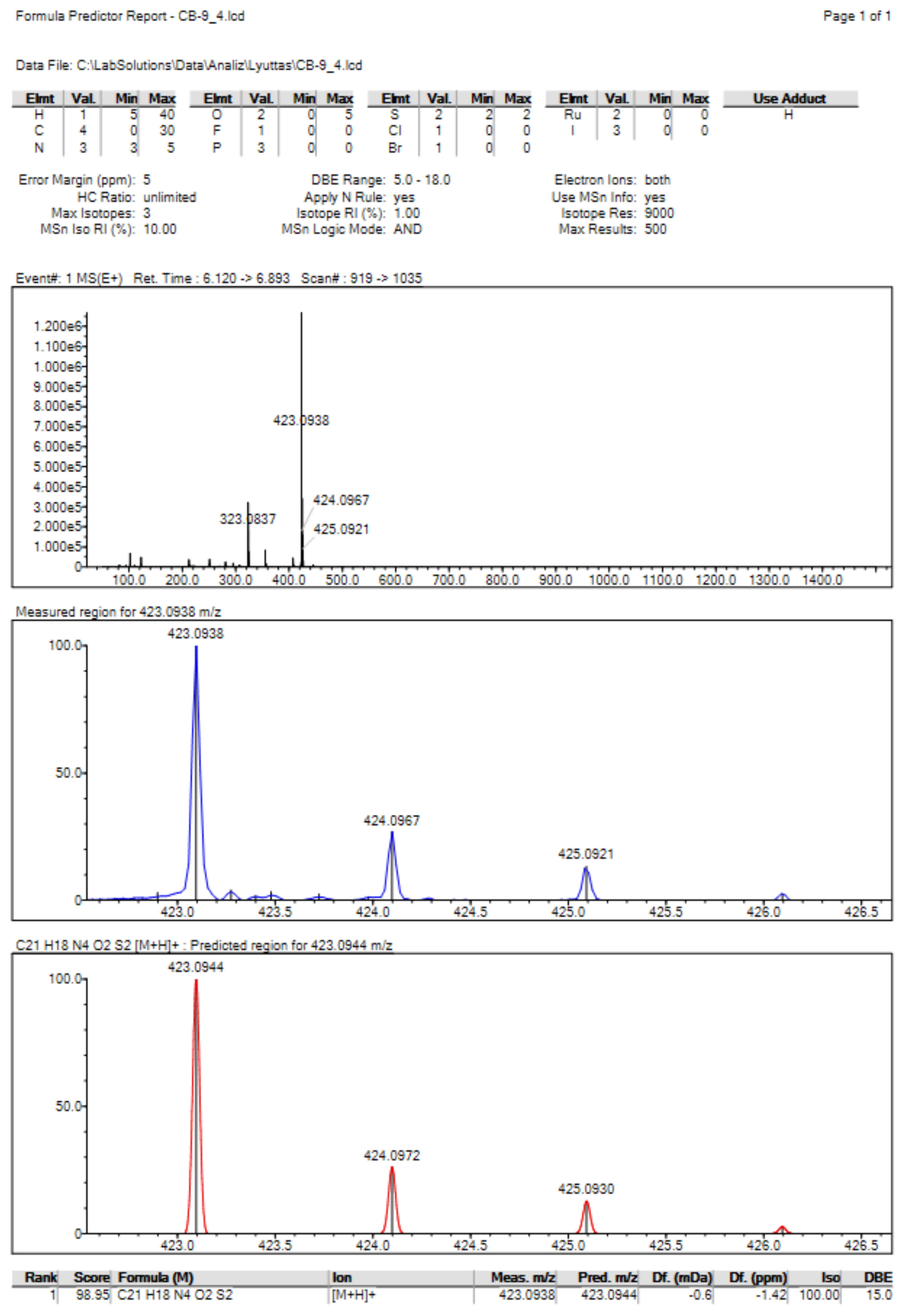

Supplement: Figure S.34 — HRMS spectrum of Compound 7 [file turkjchem-45-6-1841s34.tif]

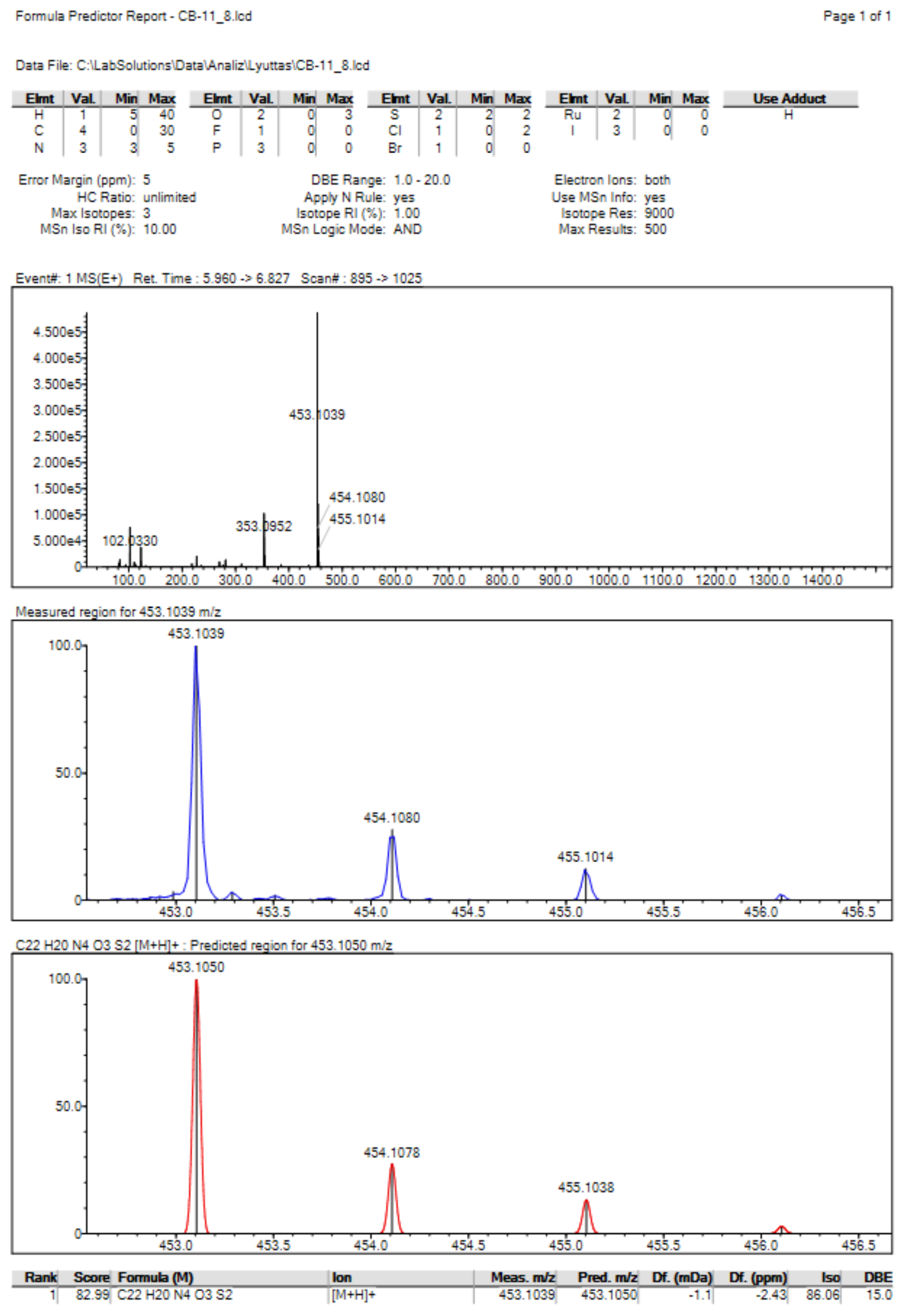

Supplement: Figure S.35 — HRMS spectrum of Compound 8 [file turkjchem-45-6-1841s35.tif]

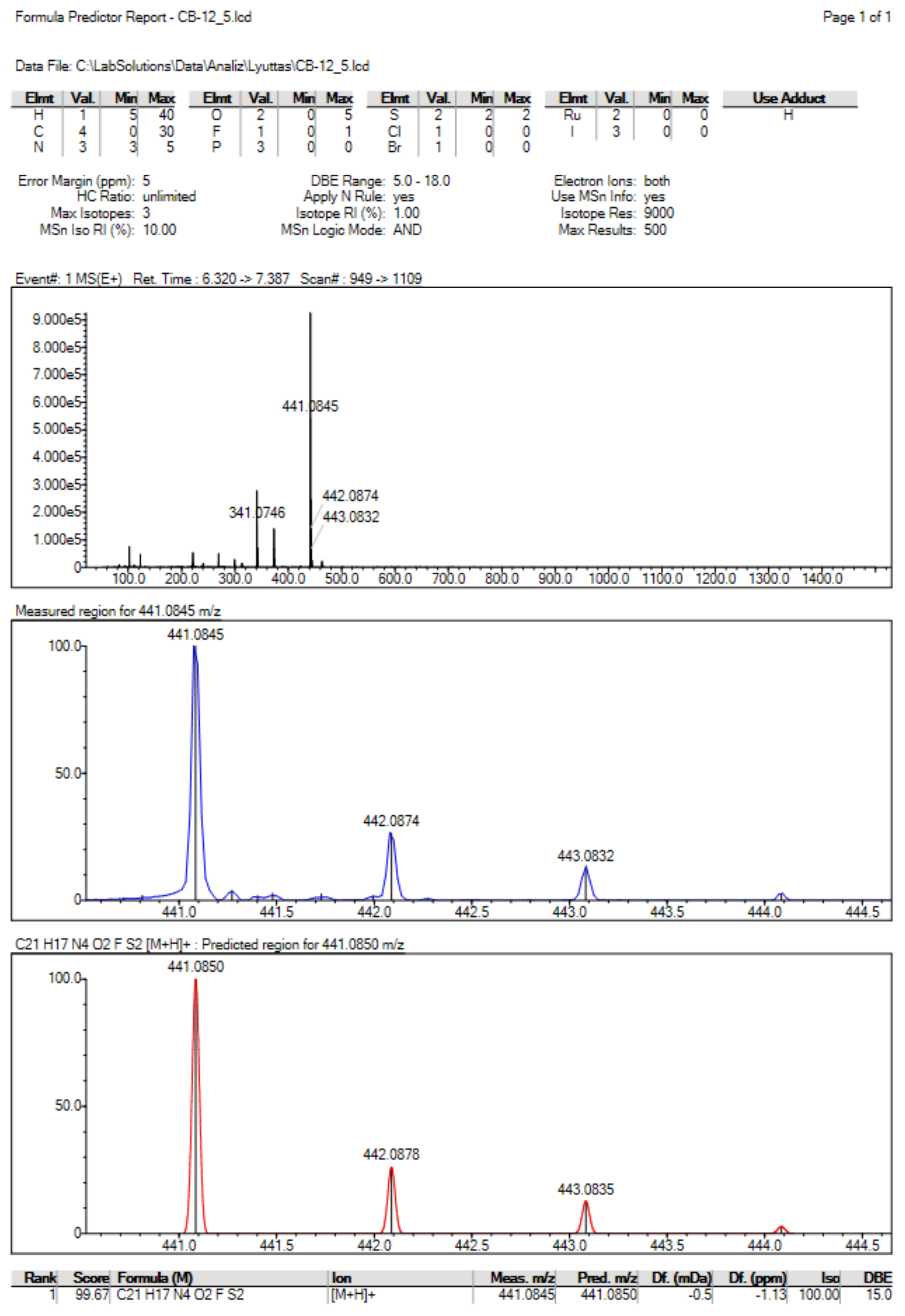

Supplement: Figure S.36 — HRMS spectrum of Compound 9 [file turkjchem-45-6-1841s36.tif]
